# Elevated catalase expression in a fungal pathogen is a double-edged sword of iron

**DOI:** 10.1371/journal.ppat.1006405

**Published:** 2017-05-22

**Authors:** Arnab Pradhan, Carmen Herrero-de-Dios, Rodrigo Belmonte, Susan Budge, Angela Lopez Garcia, Aljona Kolmogorova, Keunsook K. Lee, Brennan D. Martin, Antonio Ribeiro, Attila Bebes, Raif Yuecel, Neil A. R. Gow, Carol A. Munro, Donna M. MacCallum, Janet Quinn, Alistair J. P. Brown

**Affiliations:** 1 Aberdeen Fungal Group, MRC Centre for Medical Mycology, University of Aberdeen, Institute of Medical Sciences, Aberdeen, United Kingdom; 2 Centre for Genome-Enabled Biology and Medicine, University of Aberdeen, Aberdeen, United Kingdom; 3 Iain Fraser Cytometry Centre, University of Aberdeen, Institute of Medical Sciences, Aberdeen, United Kingdom; 4 Institute for Cell and Molecular Biosciences, University of Newcastle, Newcastle upon Tyne, United Kingdom; University of Texas-Houston Medical School, UNITED STATES

## Abstract

Most fungal pathogens of humans display robust protective oxidative stress responses that contribute to their pathogenicity. The induction of enzymes that detoxify reactive oxygen species (ROS) is an essential component of these responses. We showed previously that ectopic expression of the heme-containing catalase enzyme in *Candida albicans* enhances resistance to oxidative stress, combinatorial oxidative plus cationic stress, and phagocytic killing. Clearly ectopic catalase expression confers fitness advantages in the presence of stress, and therefore in this study we tested whether it enhances fitness in the absence of stress. We addressed this using a set of congenic barcoded *C*. *albicans* strains that include doxycycline-conditional *tetON-CAT1* expressors. We show that high basal catalase levels, rather than *CAT1* induction following stress imposition, reduce ROS accumulation and cell death, thereby promoting resistance to acute peroxide or combinatorial stress. This conclusion is reinforced by our analyses of phenotypically diverse clinical isolates and the impact of stochastic variation in catalase expression upon stress resistance in genetically homogeneous *C*. *albicans* populations. Accordingly, *cat1*Δ cells are more sensitive to neutrophil killing. However, we find that catalase inactivation does not attenuate *C*. *albicans* virulence in mouse or invertebrate models of systemic candidiasis. Furthermore, our direct comparisons of fitness *in vitro* using isogenic barcoded *CAT1*, *cat1*Δ and *tetON-CAT1* strains show that, while ectopic catalase expression confers a fitness advantage during peroxide stress, it confers a fitness defect in the absence of stress. This fitness defect is suppressed by iron supplementation. Also high basal catalase levels induce key iron assimilatory functions (*CFL5*, *FET3*, *FRP1*, *FTR1*). We conclude that while high basal catalase levels enhance peroxide stress resistance, they place pressure on iron homeostasis through an elevated cellular demand for iron, thereby reducing the fitness of *C*. *albicans* in iron-limiting tissues within the host.

## Author summary

The pathogenic yeast *Candida albicans* faces multiple challenges within its human host. These include the need to protect itself against the toxic oxidants used by the host to kill invading microbes, and the need to scavenge iron, an essential micronutrient that is limiting in certain tissues. The iron-containing enzyme, catalase, detoxifies hydrogen peroxide, thereby playing a major role in protecting *C*. *albicans* against reactive oxygen species and neutrophil killing. Indeed, we show that high basal catalase expression increases the resistance of this yeast to oxidative and combinatorial (oxidative plus cationic) stresses. Yet, rather than enhancing the virulence of *C*. *albicans* as had been predicted, high basal catalase expression decreases fungal colonisation in certain iron-limiting tissues. Furthermore, we demonstrate that catalase inactivation does not significantly perturb the virulence of *C*. *albicans* in models of systemic infection. We also show that ectopic catalase expression increases the demand for iron in *C*. *albicans*, thereby reducing the fitness of this pathogen in the absence of stress under iron-limiting conditions. Therefore, high basal catalase expression is a double-edged sword: it enhances the fitness of *C*. *albicans* in the presence of stress, but reduces fitness in the absence of stress. This explains why catalase overexpression reduces rather than enhances virulence.

## Introduction

Of the *circa* three million fungal species that are thought to inhabit our planet [1], only a relatively small number have been reported to cause infections in humans. (About 400 species are described in the *Atlas of Clinical Fungi* [2].) Nevertheless, there is an increasing awareness that these fungal pathogens impose a major burden on human health worldwide [3]. These clinically important fungi generally share common features that promote colonization of their human host, such as the thermotolerance that permits growth at body temperatures. These common features include relatively robust stress responses, which mitigate against the stresses imposed by host immune defences [e.g. 4–6]. They also include the ability to scavenge essential micronutrients, such as iron, from their host [7–10].

Iron is an essential micronutrient that is required for the functionality of key ferroproteins and haem proteins. However, excess iron is toxic because ferrous ions promote the Fenton reaction which produces highly toxic hydroxyl radicals [11], and therefore host and pathogen alike must tightly regulate their acquisition, storage and release of iron. Consequently, the levels of free ion are vanishingly low in some host niches [12]. Furthermore, following infection the host activates the process of nutritional immunity in an effort to limit iron availability for the invading microbe [10,12]. Fungal pathogens respond to this iron limitation by down-regulating genes encoding iron-containing proteins and upregulating efficient iron scavenging mechanisms [13–17]. In *Candida albicans* this response includes the induction of genes encoding ferric reductases (e.g. *CFL5*, *FRP1*), high affinity iron permeases (e.g. *FTR1*) and proteins involved in iron assimilation (e.g. *FET3*) [15]. This response allows the fungus to counter the changes in iron homeostasis within the host that are triggered by systemic candidiasis [10].

Fungal pathogens activate oxidative stress responses when they come in contact with the host [18–22], and these responses promote resistance to phagocytic attack and fungal virulence [5,23–26]. In an attempt to clear invading fungal pathogens, host neutrophils and macrophages phagocytose the fungal cells and subject them to a battery of reactive oxygen species (ROS) that damage proteins, DNA and lipids, and can induce programmed cell death [27]. The impact of ROS is augmented when combined with a cationic stress, and this synergistic impact of combinatorial oxidative and cationic stresses is thought to contribute to the potency of human neutrophils [28,29]. *C*. *albicans* cells respond to oxidative stress by inducing functions that detoxify the ROS, repair the oxidative damage, synthesize antioxidants and restore redox homeostasis. This includes the induction of genes encoding catalase (*CAT1*), superoxide dismutases (*SOD*), glutathione peroxidases (*GPX*) and components of the glutathione/glutaredoxin (*GSH1*, *TTR1*) and thioredoxin (*TSA1*, *TRX1*, *TRR1*) systems [6,30–32]. In particular, *CAT1* mRNA levels are strongly induced by oxidative stress [30,33]. However, *C*. *albicans* cells are unable to activate a normal transcriptional response to oxidative stress when subjected to combinatorial oxidative plus cationic stress or acute peroxide stress, and this contributes to the lethality of these types of stress [28,29].

Catalase (Cat1) plays a major role in protecting *C*. *albicans* against peroxide stress [28,29]. This iron-requiring enzyme, which has been well-characterised structurally [34], belongs to a superfamily of heme peroxidases and catalases that are conserved across bacteria, plants, fungi and animals [35]. Catalase catalyses the conversion of hydrogen peroxide (H_2_O_2_) to water. *C*. *albicans* cells rapidly detoxify extracellular H_2_O_2_ following exposure to an acute peroxide stress, and this detoxification is mainly dependent on catalase (*CAT1*) [28].

We showed previously that ectopic expression of catalase using the *ACT1* promoter (*ACT1*_*p*_*-CAT1*) protected *C*. *albicans* from acute oxidative and combinatorial stresses [28]. More recently, Jesus Pla’s group has confirmed that catalase overexpression protects *C*. *albicans* against peroxide stress [36]. They also demonstrated that high catalase levels provide protection against antifungal drugs. These observations raise an interesting conundrum: if catalase overexpression confers effects that might be expected to promote host colonisation, why has *C*. *albicans* not evolved to express high basal levels of catalase? We address this in this study. We show that while high basal catalase levels enhance the fitness of *C*. *albicans* in the presence of oxidative and combinatorial stresses, these high catalase levels reduce fitness in the absence of stress. We also reveal the molecular basis for this fitness defect. Our observations suggest a partial explanation for the lack of emergence of catalase overexpression during the evolution of this major fungal pathogen. We also show that, in contrast to the prevailing view [23], the virulence of *C*. *albicans* is not compromised by catalase inactivation.

## Results

### High basal catalase levels protect against oxidative and combinatorial stresses

We demonstrated previously that ectopic expression of catalase from an *ACT1* promoter-*CAT1* fusion (*ACT1*_*p*_*-CAT1*) reproducibly protected *C*. *albicans* against acute peroxide stress (5 mM H_2_O_2_) and combinatorial stress (5 mM H_2_O_2_ plus 1 M NaCl) [28]. Subsequently we noted that the stress resistance of *ACT1*_*p*_*-CAT1* cells declined over time (S1 Fig). Therefore, we constructed new *C*. *albicans* strains in which catalase expression is regulated by the doxycycline conditional *tetON* promoter [37–39]. Control strains were made by transforming congenic wild-type (*CAT1*) and catalase null strains (*cat1*Δ) with empty *tetON* vectors. Test strains were made by integrating a *tetON-CAT1* plasmid into the genome of the *cat1*Δ null mutant. We refer to these strains, which all have the same genetic background (Materials and Methods; S1 Table), as wild-type (*CAT1*), null (*cat1*Δ) and *tetON-CAT1* strains, respectively. Three isolates were generated for each strain type. For each strain type, the isolates displayed similar stress phenotypes (below).

**Fig 1:**
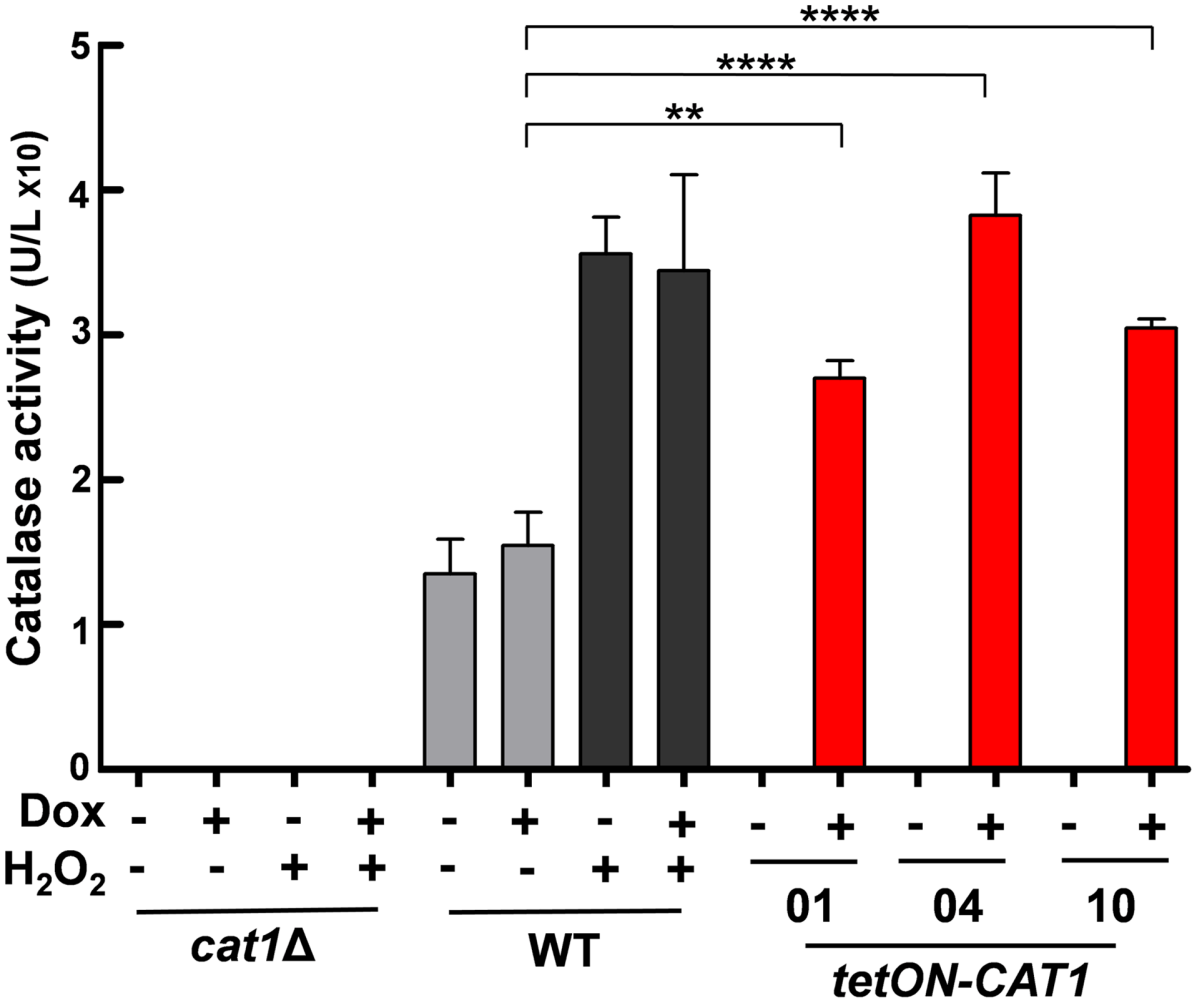
Manipulation of catalase levels in *C*. *albicans*. Catalase activities were measured in protein extracts from mid-exponential *C*. *albicans* cultures containing 0 or 20 μM doxycycline (- or + Dox, respectively): *cat1*Δ, Ca2089; wild-type, WT, Ca2084; red, *tetON-CAT1* isolates, Ca2040, Ca2043, Ca2046 (S1 Table). Immediately before harvesting, wild-type and *cat1*Δ cultures were exposed to 0 or 5 mM H_2_O_2_ for one hour. Means and standard deviations from three independent replicate experiments are shown, and the data were analysed using one-way ANOVA with Tukey’s post-hoc test: *, *p* ≤ 0.05; **, *p* ≤ 0.01; ***, *p* ≤ 0.001; ****, *p* ≤ 0.0001.

First we tested Cat1 expression levels in wild-type (*CAT1*), null (*cat1*Δ) and *tetON-CAT1* cells. Catalase levels were induced in response to oxidative stress in wild-type (*CAT1*) cells, and were undetectable in *cat1*Δ cells (Fig 1). Catalase levels in these strains were not affected by doxycycline addition. In contrast, catalase levels were strongly induced by doxycycline in *tetON-CAT1* cells (red bars, Fig 1). Significantly, wild-type cells express significant basal levels of catalase in the absence of stress (Fig 1), as we reported previously [33]. Catalase levels in doxycycline-treated *tetON-CAT1* cells were higher than these basal levels (Fig 1).

**Fig 2:**
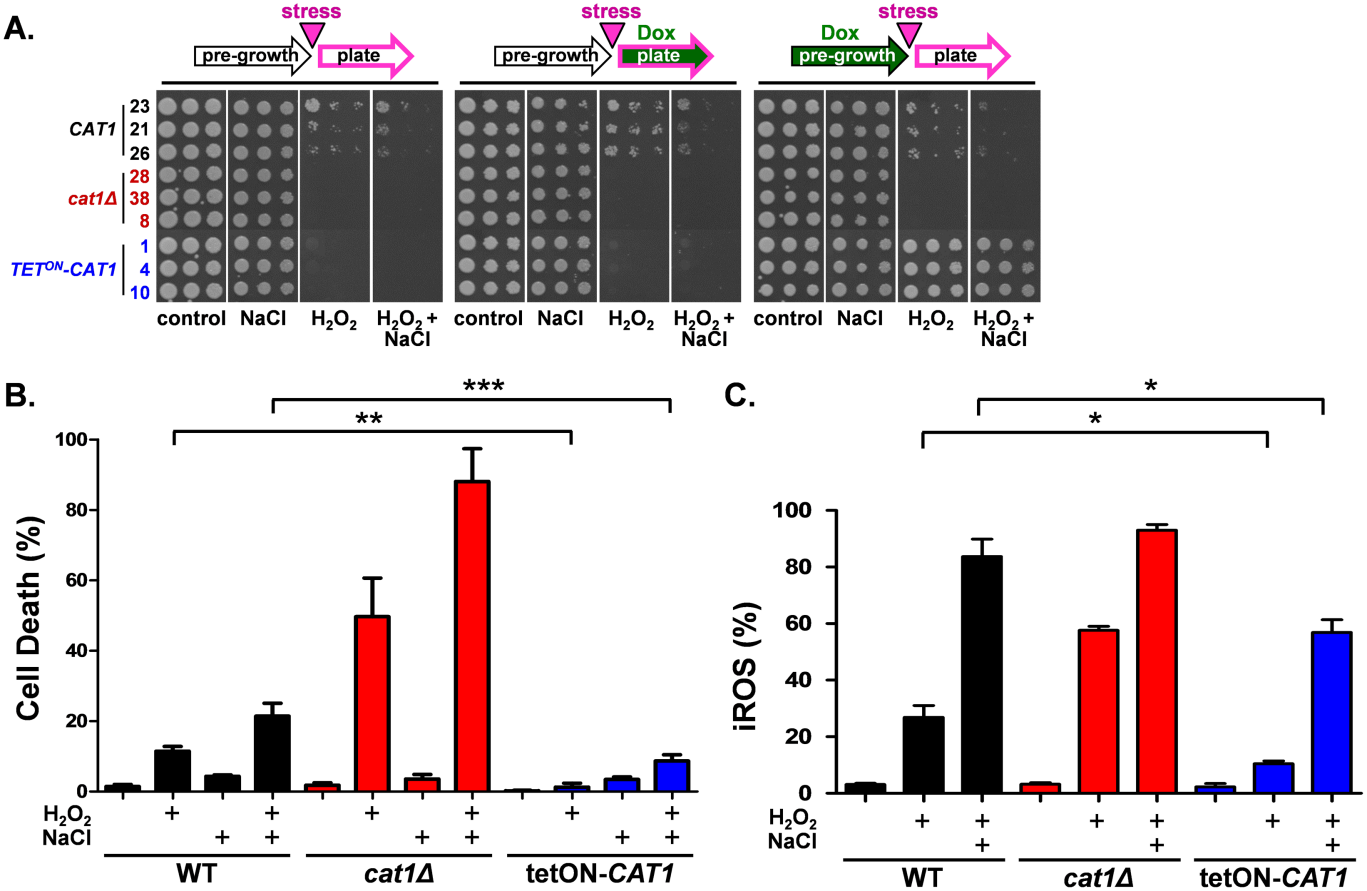
Elevated basal levels of catalase protect against oxidative and combinatorial stresses. **(A)** Dilutions of mid-exponential *C*. *albicans* cultures were spotted onto YPD plates containing different stresses and photographed after 24 h growth at 30°C: control; 1 M NaCl; 7.5 mM H_2_O_2_; or 5 mM H_2_O_2_ plus 1 M NaCl. Strains were either grown in the absence of doxycycline (left panels; see cartoons above the panels), pre-grown without doxycycline and then plated onto media containing 20 μM doxycycline (central panels), or pre-grown with 20 μM doxycycline and then plated onto media lacking doxycycline (right panels): wild-type *CAT1* isolates 23, 21, 26 (Ca2084, Ca2085, Ca2087); *cat1*Δ isolates 28, 38, 54 (Ca2089, Ca2092, Ca2030); *tetON-CAT1* isolates 1, 4, 10 (Ca2038, Ca2041, Ca2044; S1 Table). These data reflect the outputs of three independent experiments. **(B)** Mid-exponential wild type (*CAT1*, *Ca*2084), *cat1*Δ (Ca2089) and *tetON-CAT1* cells (Ca2038) grown in YPD containing 20 μM doxycycline were treated with 5 mM H_2_O_2_ and/or 1 mM NaCl for 1 h. Cells were stained with propidium iodide (PI) and the percentage of PI positive (dead) cells quantified by flow cytometry. Means and standard deviations from three replicates are presented: *, *p* ≤ 0.05; **, *p* ≤ 0.01; ***, *p* ≤ 0.001. **(C)** Wild type (*CAT1*, Ca2084), *cat1*Δ (Ca2089) and *tetON-CAT1* cells (Ca2038) grown in YPD plus 20 μM doxycycline were treated with 5 mM H_2_O_2_ and/or 1 mM NaCl for 1 h, and their accumulation of intracellular ROS quantified by flow cytometry. Means and standard deviations from three replicates are presented: *, *p* ≤ 0.05.

We then compared the stress resistance of wild-type, null and *tetON-CAT1* cells (Fig 2A & S1 Fig). As expected [23,28,40], wild-type (*CAT1*) cells displayed modest resistance to an oxidative stress (H_2_O_2_) and a combinatorial stress (H_2_O_2_ plus NaCl), whereas the null mutant (*cat1*Δ) was sensitive to both types of stress. These phenotypes were not affected by the presence or absence of doxycycline (Fig 2A). In the absence of doxycycline, the *tetON-CAT1* strains were sensitive to both oxidative and combinatorial stress, reflecting their null *cat1*Δ background. When these strains were pre-grown with doxycycline, they displayed enhanced oxidative and combinatorial stress resistance (Fig 2A). This reinforces our earlier conclusion [28] that elevated basal *CAT1* expression levels protect *C*. *albicans* cells against a sudden and acute oxidative or combinatorial stress.

Interestingly, the *tetON-CAT1* strains were sensitive to both oxidative and combinatorial stress when pre-grown in the absence of doxycycline and the inducer was only provided when the stress was imposed (Fig 2A). We then measured the impact of individual and combinatorial oxidative (H_2_O_2_) and cationic (NaCl) stresses upon cell death by cytometric analysis of propidium iodide (PI) stained doxycycline-grown cell populations (Fig 2B). Relative to the control wild-type strain, *cat1*Δ null cells were more sensitive, and *tetON-CAT1* cells were more resistant to these oxidative and combinatorial stresses. Compared to the control wild type cells, doxycycline-treated *tetON-CAT1* cells displayed 9-fold less cell death following exposure to the oxidative stress, and 2.5-fold less death after the combinatorial stress (Fig 2B).

This correlated with a reduction in internal ROS accumulation following stress imposition by *tetON-CAT1* cells relative to the wild-type and *cat1*Δ cells (Fig 2C). The accumulation of intracellular ROS was 2.6-fold lower in doxycycline-treated *tetON-CAT1* cells after the peroxide stress, and 1.5-fold lower following the combinatorial stress, compared to the wild type control (Fig 2C). Taken together, our data indicate that cells with low catalase levels at the point of stress imposition are more sensitive to peroxide than cells with high catalase levels. This suggests if catalase levels are low at the point of stress imposition, the dynamics of catalase induction are too slow to permit the normally rapid clearance of peroxide [28] and to prevent ROS-mediated cell death [27]. The data indicate that *C*. *albicans* cells require high basal levels of catalase *at the time of stress imposition* if they are to survive an acute oxidative or combinatorial stress.

### Oxidative stress resistance and catalase levels in clinical isolates

**Fig 3:**
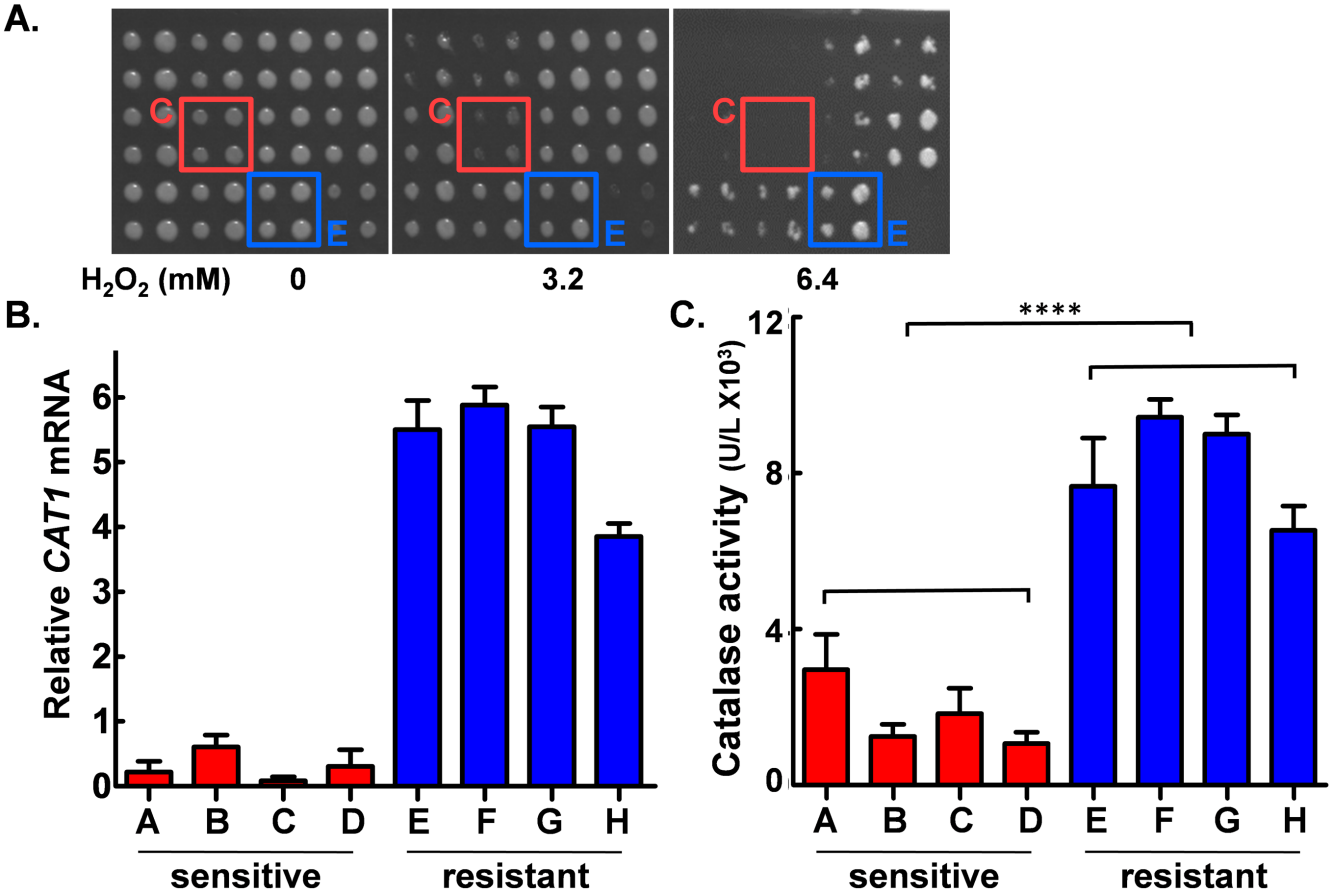
Basal catalase expression and oxidative stress resistance in *C*. *albicans* clinical isolates. **(A)** A subset is shown of the sixty-five clinical isolates that were robotically screened (duplicate spots for two dilutions) on YPD plates containing 0, 3.2 or 6.4 mM H_2_O_2_. A relatively H_2_O_2_ sensitive (C, red) and resistant strains (E, blue) are highlighted. **(B)** Four relatively sensitive strains (A-D, red), and three relatively resistant strains plus a control laboratory strain (E-H, blue) were selected, and their basal *CAT1* mRNA levels during growth on YPD in the absence of stress measured by qRT-PCR relative to the internal *ACT1* mRNA control: A, 81/064; B, AM2003/0025; C, AM2005/0377; D, SCSBB417709; E, J990102; F, IHEM16614; G, SC5314; H, CAI4+CIp10 (S1 Table). **(C)** Basal catalase levels in the same strains were assayed under the same growth conditions as (B). Means and standard deviation from three replicates are presented, and the difference between the sets of sensitive and resistance strains compared statistically: ****, *p* ≤ 0.0001.

*C*. *albicans* clinical isolates display a high degree of natural variation [41,42]. We exploited this to select strains that display relatively low levels of oxidative stress resistance. A diverse range of *C*. *albicans* clinical isolates (65 in total) from different epidemiological clades and from different patient colonisation sites were subjected to a robotic screen in which they were plated on YPD containing different peroxide concentrations (Fig 3A). All of the isolates tested displayed a degree of resistance to this stress, showing some growth at 3.2 mM H_2_O_2_. However, some isolates were more resistant to peroxide, displaying robust growth at 6.4 mM H_2_O_2_, whereas sensitive strains were unable to grow at this H_2_O_2_ concentration. We selected a subset of four sensitive isolates and three resistant isolates (which included SC5314, the clinical isolate from which most laboratory strains are derived), and compared the basal *CAT1* expression levels in these isolates to a standard laboratory strain (CAI4 containing CIp10 (*URA3*)). Basal *CAT1* mRNA levels were lower in the oxidative stress sensitive isolates tested (Fig 3B), and furthermore, the basal levels of the enzyme were also lower in these isolates (Fig 3C). These data are consistent with the idea that elevated basal catalase levels promote oxidative stress resistance in *C*. *albicans*.

### Population heterogeneity promotes *C*. *albicans* survival following oxidative stress

**Fig 4:**
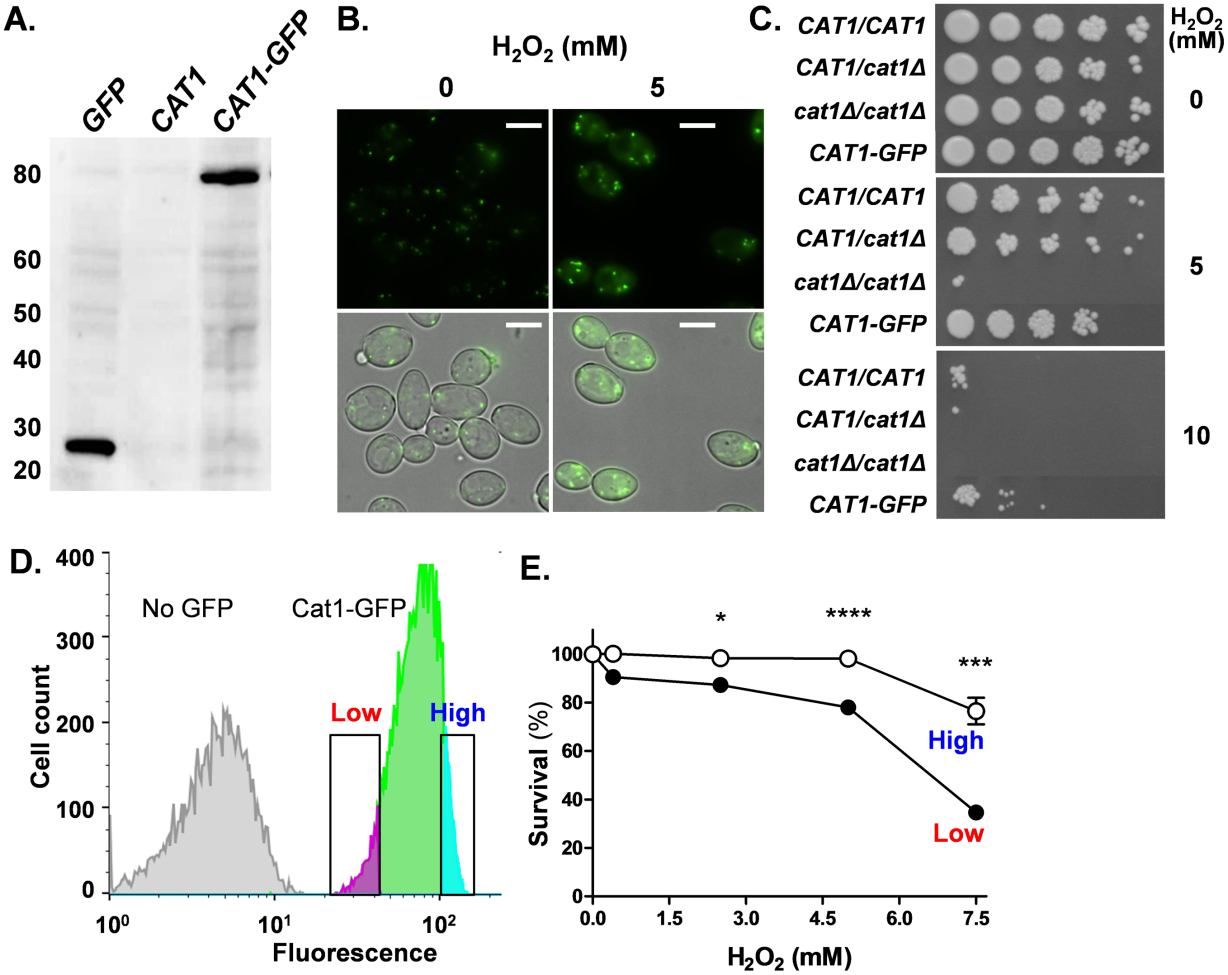
Stochastic differences in catalase expression within a population of *C*. *albicans* cells affect resistance to peroxide stress. **(A)** Western blot of GFP in *C*. *albicans* cells grown in YPD at 30°C: GFP, cells expressing GFP from pACT1-GFP (Ca230); *CAT1*, control cells with no GFP (Ca674); *CAT1-GFP*, cells expressing Cat1-GFP (Ca2213) (S1 Table). **(B)** DIC and fluorescence microscopy of log-phase *CAT1-GFP* cells (Ca2213) exposed to 0 or 5 mM H_2_O_2_ for 1 h: scale bar = 5 μm. **(C)** The resistance of *C*. *albicans* strains to peroxide was assessed by plating onto YPD plates containing 0, 5 or 10 mM H_2_O_2_: *CAT1/CAT1*, Ca674; *CAT1/cat1*Δ, Ca1862; *cat1*Δ/*cat1*Δ, Ca1864; *CAT1-GFP*, Ca2213. For each stress condition, the images for all strains were taken from the same plate. **(D)** Sorting of *C*. *albicans* cells of similar size expressing relatively low or high levels of Cat1-GFP within the same population growing on YPD (no stress) by FACS: Cat1-GFP cells, Ca2213; No GFP control, Ca674. **(E)** The survival of FACS sorted cells with low or high Cat1-GFP levels on YPD plates containing different concentrations of H_2_O_2_ (CFU) expressed as a percentage of the survival for the no stress control. Means and standard deviations from three replicates are presented: *, *p* ≤ 0.05; **, *p* ≤ 0.01; ***, *p* ≤ 0.001; ****, *p* ≤ 0.0001.

Next we examined how a subset of cells within an apparently homogeneous population of *C*. *albicans* cells can survive an acute oxidative stress [28,36,43]. Based on the above observations, we reasoned that this might be partly explained by stochastic variation in basal catalase levels between individual cells in such a population. To test for potential population heterogeneity in basal catalase levels we generated a strain in which both *CAT1* alleles were tagged with GFP (*CAT1-GFP/ CAT1-GFP*) to express a Cat1-GFP fusion protein. Western blotting revealed a Cat1-GFP protein of the expected mass in these cells (approximately 80 kDa: Fig 4A), and the GFP fluorescence was located in punctate spots (Fig 4B), consistent with the peroxisomal localisation of catalase in *C*. *albicans* [44]. We then compared the oxidative stress resistance of the *CAT1-GFP* strain with congenic control wild-type (*CAT1/CAT1*), heterozygous (*CAT1/ cat1*Δ) and null (*cat1*Δ/*cat1*Δ) strains. The *CAT1-GFP* strain was as resistant to oxidative stress as the wild-type control (Fig 4C), indicating that the *CAT1-GFP* alleles are functional.

We then examined the basal levels of GFP fluorescence in unstressed populations of exponentially growing *C*. *albicans CAT1-GFP* cells by flow cytometry. As predicted, these genetically homogeneous cell populations displayed heterogeneity with respect to their Cat1-GFP expression levels (Fig 4D & S2 Fig). Using flow cytometry, we selected cells of similar size, sorted cells that display relatively low levels of Cat1-GFP from those expressing high levels (Fig 4D & S2 Fig), and then plated them onto media containing a range of H_2_O_2_ concentrations. Cells expressing relatively high levels of Cat1-GFP were more resistant to peroxide stress (Fig 4E). When an analogous experiment was performed with cells expressing a control gene (*ACT1-GFP*), stochastic differences in *ACT1-GFP* expression did not affect oxidative stress resistance (S3 Fig). These observations reinforce our conclusion that high *basal* levels of catalase promote oxidative stress resistance. Furthermore, this confirms that *C*. *albicans* cell populations display stochastic variation in their basal *CAT1* expression levels, and that this contributes to the survival of a subset of *C*. *albicans* cells following an acute oxidative stress.

### Impact of catalase levels on host colonisation during systemic infection

**Fig 5:**
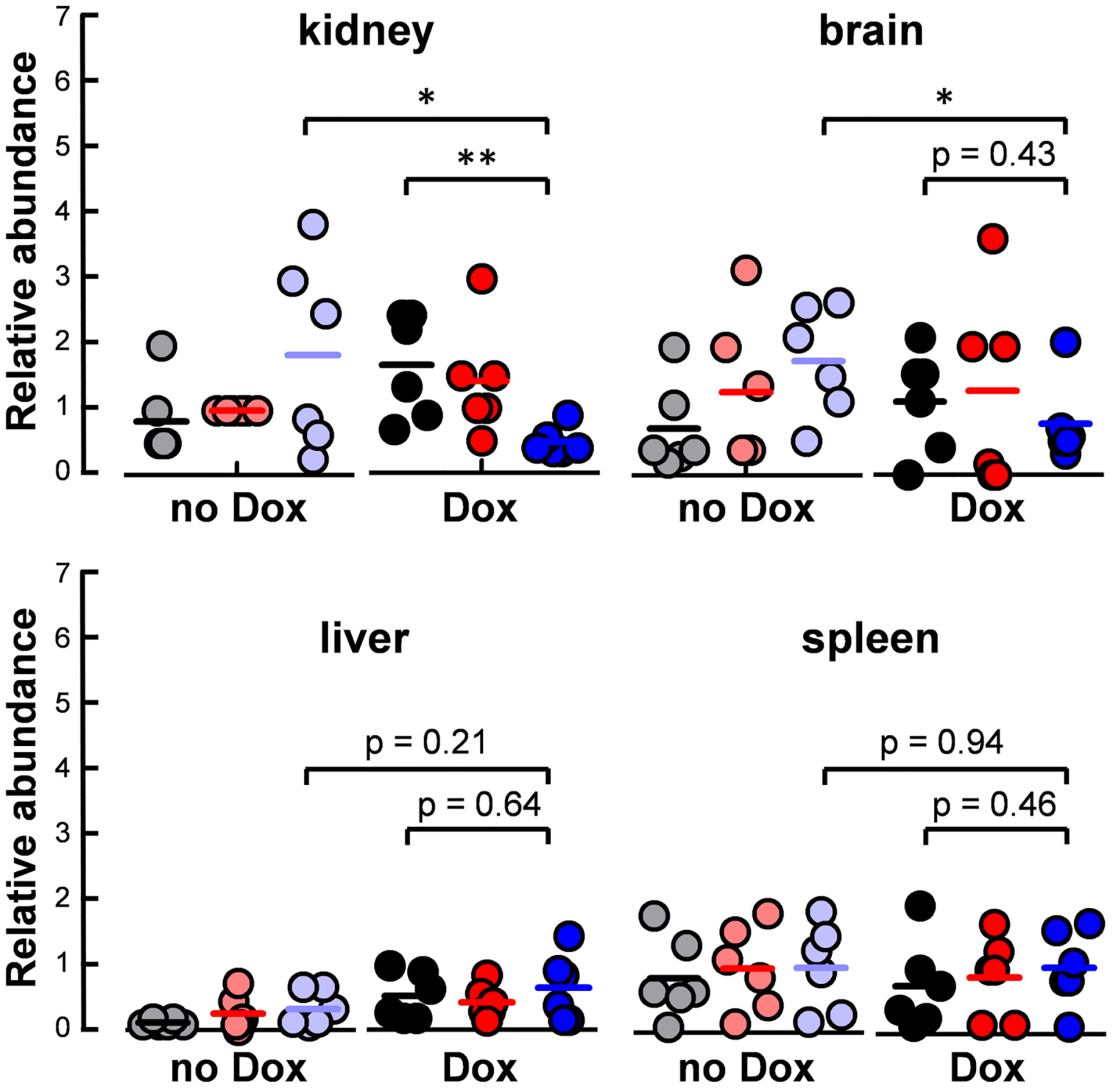
Impact of catalase levels on host colonisation during systemic infection. The nine *C*. *albicans* strains were grown separately in medium containing 0 or 20 μM doxycycline, mixed in approximately equal proportions, and then this pool of nine barcoded strains used to initiate systemic infection in mice via the tail vein (n = 6 mice per group): wild-type (*CAT1*) strains 23, 21, 26 (Ca2084, Ca2085, Ca2087); *cat1*Δ strains 28, 38, 54 (Ca2089, Ca2092, Ca2030); *tetON-CAT1* strains 1, 4, 10, (Ca2038, Ca2041, Ca2044; S1 Table). Mice that received the pool of nine *C*. *albicans* strains pre-grown with doxycycline were treated with doxycycline, whereas mice that were infected with the pool of *C*. *albicans* strains pre-grown without doxycycline did not (Materials and Methods). After four days, mice were killed by cervical dislocation and the kidneys, brains, livers and spleens harvested. The fungal cells infecting these organs were grown on YPD plates, genomic DNA isolated from each fungal population, and the abundance of each barcode in each fungal population was quantified by barseq. The Relative Abundance of each barcode in each tissue from each mouse was calculated relative to the total number of barcode reads for that specific sample, and then normalised against the proportion for that barcode in the pool of nine *C*. *albicans* strains used to infect that mouse. Each symbol represents data for a single mouse. The pairwise comparisons indicated were analysed using the Students t-test: *, *p* ≤ 0.05; **, *p* ≤ 0.01. Data are presented for three of the nine barcoded *C*. *albicans* strains: *CAT1* strain 23, grey and black; *cat1*Δ strain 28, pink and red; *tetON-CAT1* strain 1, pale blue and blue; no doxycycline, grey, pink, pale blue; 20 μM doxycycline, black, red, blue. These wild type and *cat1*Δ strains were representative of the other isolates. However, *tetON-CAT1-*1 behaved differently from the other *tetON-CAT1* isolates, which were shown subsequently to have lost their phenotype (below).

We tested whether high basal catalase levels affect the ability of *C*. *albicans* to colonise different tissues during systemic infection. At first we reasoned that the elevated oxidative stress resistance conferred by high basal catalase levels (above) might enhance host colonisation. To test this we compared directly the three isolates for wild-type (*CAT1*), null (*cat1*Δ) and *tetON-CAT1* strains (nine in total) using a barcode sequencing (barseq) strategy. The *C*. *albicans* strains were pre-grown separately in the presence or absence of doxycycline. Approximately equal amounts of the nine doxycycline-treated strains were mixed and used to induce disseminated candidiasis in doxycycline-treated mice. In parallel, the nine untreated control *C*. *albicans* strains were mixed and used to infect untreated mice. Mice from each group were culled after four days, and the fungal cells harvested from their kidneys, livers, spleens and brains. Barseq was then performed on genomic DNA from these fungal populations to determine the relative proportion of each *C*. *albicans* strain in each tissue. We observed significant differences between the wild-type (*CAT1*) and *tetON-CAT1* strains in their ability to colonise certain tissues (Fig 5). Doxycycline-treated *tetON-CAT1* cells were less able to colonise the kidney and brain than the control untreated *tetON-CAT1* cells, but this was not the case in the liver and spleen. This effect was observed for *tetON-CAT1-*1 cells, but not for the other two *tetON-CAT1* isolates (4 and 10: S1 Table). This correlated with a reduction in catalase levels in these isolates (S4A Fig) and a corresponding loss of phenotype (S4B Fig). Therefore, like *ACT1*_*p*_*-CAT1* cells (above; S1 Fig), isolates 4 and 10 appeared to have lost their phenotype over time. Taken together, our data indicate that, contrary to our initial prediction, high basal catalase expression levels appear to compromise, rather than enhance, the ability of *C*. *albicans* to colonise certain tissues.

To our surprise, we did not observe any significant differences between the wild-type (*CAT1*) and null (*cat1*Δ) strains in their ability to colonise the host (Fig 5). All of the wild-type and null isolates displayed similar levels of colonisation. This indicated that cells lacking catalase can infect the host—a conclusion that contrasts with the prevailing view that *C*. *albicans cat1*Δ null cells display attenuated virulence [23,40]. We reasoned that *cat1*Δ cells might be able to colonise host tissues if they are co-infected with *CAT1* and *tetON-CAT1* cells. For example, *cat1*Δ null cells might be rescued via a “cheater” or “bystander” effect [45,46], whereby catalase expressing cells protect the null mutant against local peroxide stress.

**Fig 6:**
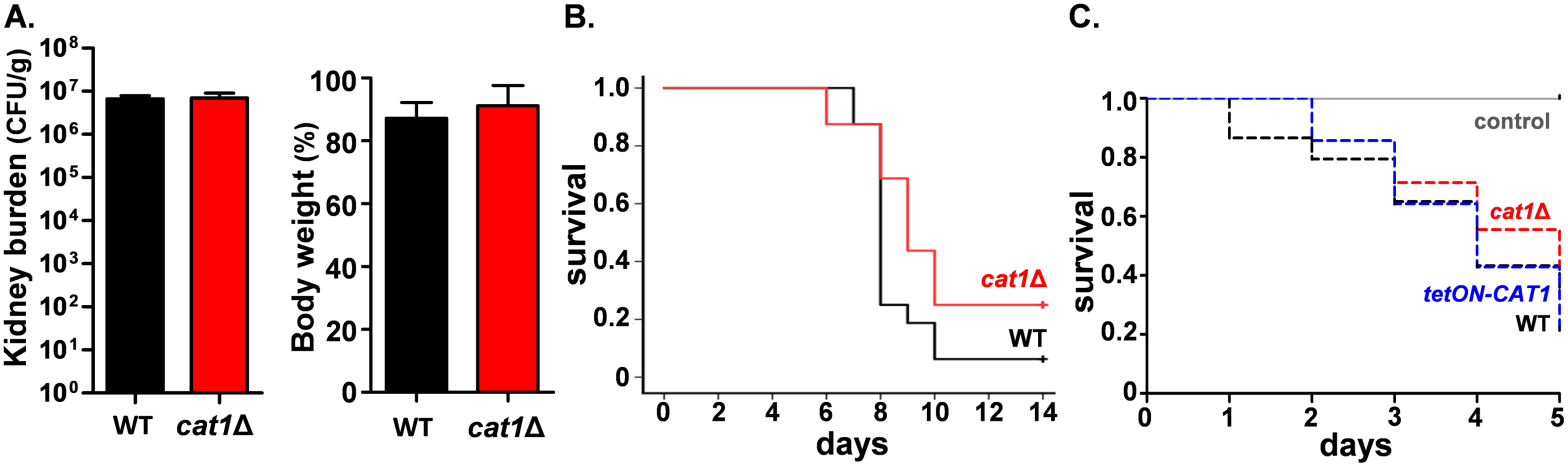
Inactivation of catalase does not attenuate *C*. *albicans* virulence. **(A)** The virulence of *C*. *albicans* wild-type (*CAT1*, Ca2084) and *cat1*Δ (Ca2089) strains was first tested in a short term model of systemic infection. Mice were infected with similar doses of either *CAT1* or *cat1*Δ cells via their tail vein, and their weights monitored over time. Animals were sacrificed after four days, their weight change determined and their kidney fungal burdens assayed. **(B)** The same strains were compared in a longer term mouse model of systemic infection. Mouse survival data are presented in a Kaplan-Meier plot: the differences are not statistically significant (*p* = 0.074). **(C)** *C*. *albicans* wild-type (*CAT1*, Ca2084), *cat1*Δ (Ca2089) and *tetON-CAT1* strains (Ca2038) were compared in the *Galleria mellonella* model of systemic infection. As a negative control, larvae were injected with phosphate buffered saline. Survival data are presented in a Kaplan-Meier plot. The differences between the *C*. *albicans* strains are not statistically significant (*p* = 0.68).

We tested this by comparing the virulence of our wild-type (*CAT1*) and null (*cat1*Δ) strains separately in the three-day murine model of systemic candidiasis [47]. We observed no significant difference between the wild-type or mutant strains with respect to their fungal burdens in the kidneys, and the strains induced similar levels of weight loss in mice, yielding similar outcome scores that displayed no significant difference (Fig 6A). This observation reinforced the idea that inactivating *CAT1* does not attenuate the virulence of *C*. *albicans*.

Wysong and co-workers observed a virulence defect for *cat1*Δ cells using a long-term mouse model of systemic candidiasis [23]. Therefore, it seemed possible that our short-term and their long-term model of systemic infection might yield differing outcomes for *C*. *albicans cat1*Δ cells. To test this we re-examined the virulence of our wild-type (*CAT1*) and null (*cat1*Δ) strains in mice over 14 days. (We were unable to access the strains used by Wysong and co-workers [23]. Hence we could not perform a direct comparison with their mutant.) No major difference in the virulence of wild-type and *cat1*Δ cells was observed using a long term infection model (p = 0.074: Fig 6B). We also compared our wild-type (*CAT1*), null (*cat1*Δ) and *tetON-CAT1* strains in *Galleria mellonella*, observing no significant difference in their virulence in this invertebrate model of systemic candidiasis (p = 0.68: Fig 6C).

The *cat1*Δ mutant generated by Wysong and co-workers had the *URA3* marker inserted at the *cat1* locus (*cat1*::*URA3*) [23]. In contrast, in our *cat1*Δ mutant *URA3* was reintroduced at the *RPS1* locus using the CIp10 plasmid backbone [48]. After the study of Wysong and co-workers was published [23], *URA3* position effects were found to influence *C*. *albicans* virulence, and reinsertion of *URA3* at *RPS1* using CIp10 was shown to overcome these effects [49]. We conclude that *CAT1* inactivation does not significantly attenuate the virulence of *C*. *albicans*.

### Catalase promotes resistance to neutrophil killing

**Fig 7:**
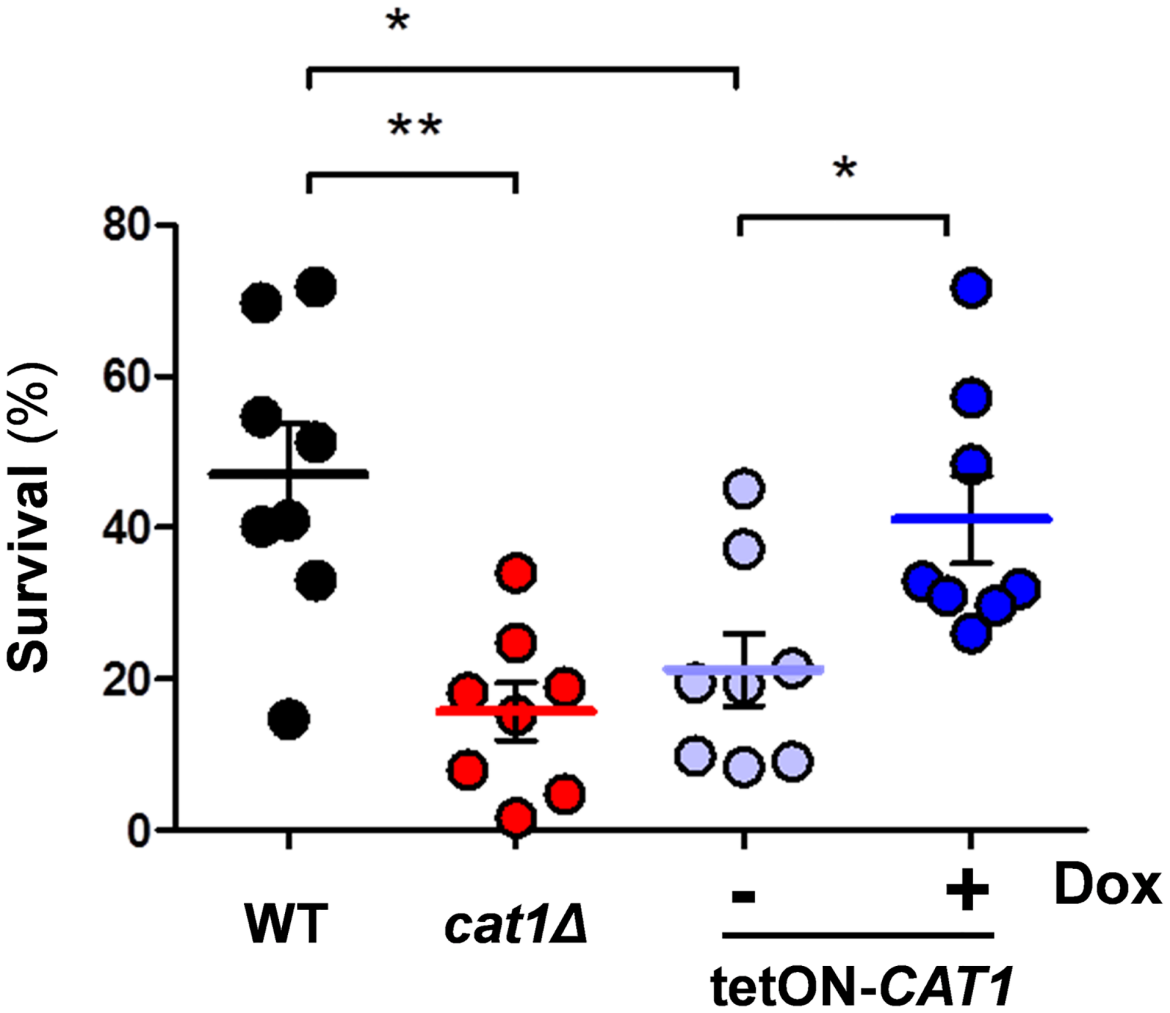
Impact of catalase on resistance to neutrophil killing. *C*. *albicans* wild-type (*CAT1*, black, Ca2084), *cat1*Δ (red, Ca Ca2089) and *tetON-CAT1* cells (Ca2038) pre-grown with 0 or 20 with μM doxycycline (pale blue and blue, respectively) were exposed to human neutrophils for 2 h, and then fungal survival assayed. Each data point represents the mean for three replicates from one healthy donor. The data were analysed using one-way ANOVA with Tukey’s post-hoc test: *, *p* ≤ 0.05; **, *p* ≤ 0.01.

It has been reported that catalase null mutants do not display significantly higher sensitivities to neutrophil killing [5]. Once again, these experiments were performed with a *cat1Δ* null mutant in which *URA3* was integrated at the *CAT1* locus (*cta1Δ*::*loxP-URA3-loxP*: [5]). Therefore, in light of our findings (above), we retested neutrophil killing using our new *cat1Δ* strain in which *URA3* is integrated at the *RPS1* locus. We tested the strains separately to exclude potential cheater effects [45,46]. We observed that, following exposure to human neutrophils, our new *cat1Δ* strain displayed significantly reduced survival compared to the congenic wild-type control (Fig 7). This strengthens the observation of Miramon and co-workers, who reported a slight difference between *cat1Δ* and *CAT1* cells that was not statistically significant [5]. Furthermore, we also observed a statistically significant difference in neutrophil killing between *tetON-CAT1* cells that were pre-grown in the presence or absence of doxycycline (Fig 7). These data indicate that catalase promotes the resistance of *C*. *albicans* against neutrophil attack. We note that elevated basal levels of catalase did not enhance the resistance of *C*. *albicans* to neutrophil killing in our hands (Fig 7: compare wild type and doxycycline-treated *tetON-CAT1* cells).

### Elevated catalase levels affect the ability of *C*. *albicans* to compete *in vitro*

**Fig 8:**
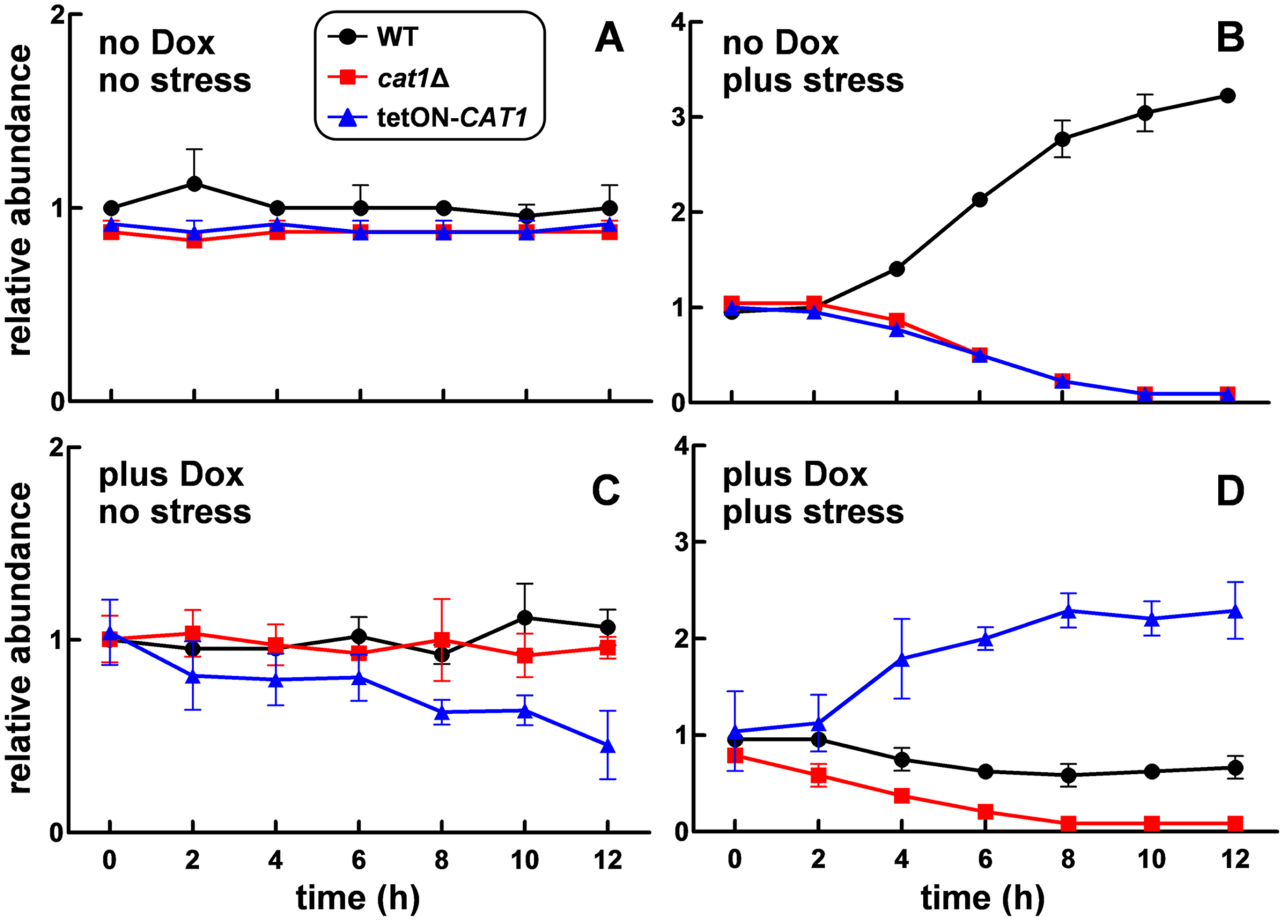
Impact of catalase levels upon *C*. *albicans* fitness in the presence or absence of peroxide stress. The nine barcoded *C*. *albicans* wild-type, *cat1*Δ and *tetON-CAT1* strains (S1 Table) were pre-grown separately in YPD containing 0 or 20 μM doxycycline. The cells grown in the presence of doxycycline were then mixed in roughly equal proportions and used to inoculate fresh YPD cultures containing doxycycline (plus Dox). In parallel, the cells grown in the absence of doxycycline were mixed and used to inoculate fresh YPD cultures lacking doxycycline (no Dox). Parallel cultures contained 0 or 5 mM H_2_O_2_ (no stress and plus stress, respectively). Cells were harvested at various time points over a 12 h period, and barseq was performed on genomic DNA extracted from each sample. The relative abundance of each barcode in each sample was then calculated relative to its starting abundance. Data are shown for one barcoded strain of each type (means and standard deviation from three replicate measurements): wild-type (*CAT1*), black, Ca2084; *cat1*Δ, red, Ca2089; *tetON-CAT1*, blue, Ca2038. **(A)** Competition between wild-type, *cat1*Δ and *tetON-CAT1* strains in the absence of doxycycline and the absence of peroxide stress. **(B)** Competition in the absence of doxycycline and the presence of stress. **(C)** Competition in the presence of doxycycline and the absence of stress. **(D)** Competition in the presence of both doxycycline and stress.

*C*. *albicans cat1*Δ cells are clearly sensitive to oxidative stress (Fig 2). However, in mixed populations they could conceivably be rescued by catalase expressing cells. Therefore, we tested whether *cat1*Δ cells act as cheaters by examining their fitness in mixed cultures alongside wild-type (*CAT1*) and *tetON-CAT1* cells. The three barcoded for the null mutant, wild-type and *tetON-CAT1* strains were pre-grown separately in the presence of doxycycline, mixed in approximately equal proportions, and then used to inoculate YPD cultures containing doxycycline. A parallel mixture of untreated barcoded strains was also prepared, and this untreated mixture used to inoculate YPD cultures without doxycycline. The relative fitness of each strain was then compared in the presence or absence of oxidative stress (5 mM H_2_O_2_), by comparing the relative abundance of each barcode over time in each culture by barseq. With one notable exception (discussed below), the three isolates for each strain type displayed similar behaviours (Fig 8).

In the absence of doxycycline and stress, the relative abundance of the wild-type (*CAT1*), null (*cat1*Δ) and *tetON-CAT1* strains did not change significantly over the twelve hour period examined (Fig 8A). In contrast, in the absence of doxycycline but in the presence of stress, the abundance of *cat1*Δ and *tetON-CAT1* cells rapidly declined in the population and these strains were rapidly outcompeted by the wild-type *CAT1* strains (Fig 8B). The comparable behaviour for the *cat1*Δ the *tetON-CAT1* cells under these conditions was entirely consistent with the negligible catalase levels in *tetON-CAT1* cells without doxycycline induction (Fig 1). These data strongly reinforce the view that catalase is vital for peroxide stress resistance in *C*. *albicans* [5,23,33,36,40]. Our data also show that *cat1*Δ cells do not act as cheaters: they are not rescued by catalase expressing cells under peroxide stress conditions (Fig 8B).

In the presence of doxycycline in the presence of stress, the *tetON-CAT1* cells rapidly outcompeted the null (*cat1*Δ) cells (Fig 8D). This again highlighted the peroxide sensitivity of *cat1*Δ cells. Significantly, the *tetON-CAT1* cells also out-competed wild-type (*CAT1*) cells (Fig 8D), confirming directly that ectopic catalase expression enhances oxidative stress resistance (Fig 2) [28,36]. Therefore, elevated basal catalase levels increase the fitness of *C*. *albicans* cells in the presence of peroxide stress.

Interestingly, in the presence of doxycycline but in the absence of peroxide stress, there was a decrease in the abundance of *tetON-CAT1-*01 cells in the population over the twelve hour time-course, relative to the wild-type (*CAT1*) and null (*cat1*Δ) cells (Fig 8C). This suggested that ectopic *CAT1* expression might render *C*. *albicans* cells less fit in the absence of stress.

### Ectopic catalase expression confers a fitness defect in the absence of stress that is suppressed by iron supplementation

**Fig 9:**
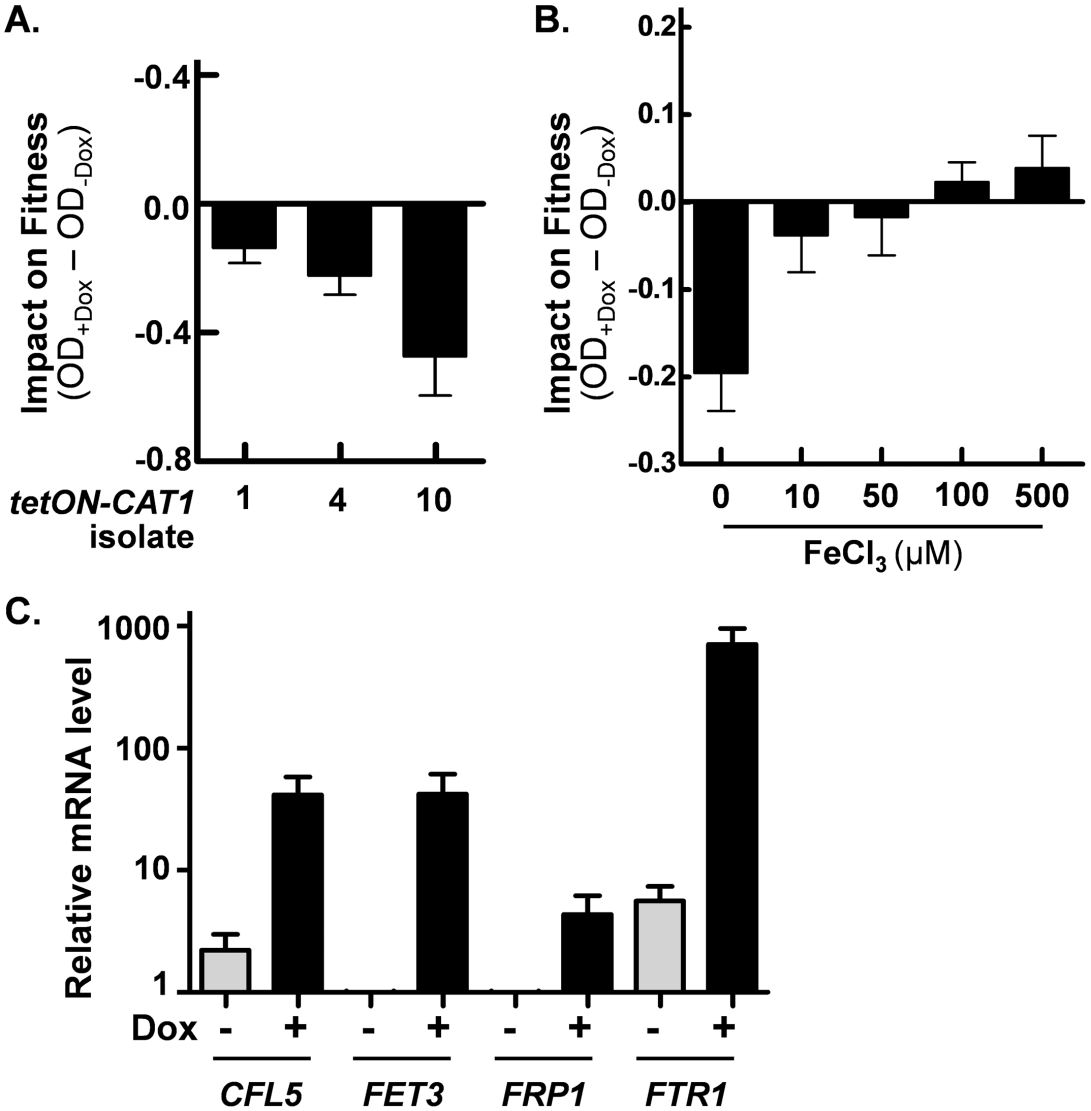
In the absence of stress, elevated catalase levels impose a fitness defect on *C*. *albicans* that is suppressed by iron supplementation. **(A)** The growth of *C*. *albicans tetON-CAT1* isolates (1, 4, 10) was monitored (OD_600_) in YPD containing 0 or 20 μM doxycycline (Ca2040, Ca2043, Ca2046; S1 Table). The impact of *tetON-CAT1* induction upon fitness was assayed by subtracting the OD after growth in the absence of doxycycline (OD_-Dox_) from the OD in the presence of doxycycline (OD_+Dox_). Data represent the means and standard deviations from three independent experiments. **(B)** The impact of iron on the fitness of *C*. *albicans tetON-CAT1* strain 1 (Ca2040) was measured in YPD cultures containing 0 or 20 μM doxycycline plus different concentrations of FeCl_3_. Data represent the means and standard deviations from three independent experiments. **(C)** The effect of *tetON-CAT1* expression on genes involved in iron assimilation and homeostasis was assessed by qRT-PCR of specific transcripts (relative to the *ACT1* mRNA internal control) during growth of *C*. *albicans tetON-CAT1* isolate 1 (Ca2040) in YPD containing 0 or 20 μM doxycycline. The data, which represent the means and standard deviations from three replicate measurements, are normalised relative to the corresponding transcript level in wild type cells grown with doxycycline.

Doxycycline-treated *C*. *albicans tetON-CAT1* cells appeared to display a fitness defect in the absence of stress (Fig 8C). We tested this further by examining biomass formation on YPD (final OD_600_) (Fig 9A). All of the strains displayed similar growth in the absence of doxycycline, and the wild-type (*CAT1*) controls remained unaffected by doxycycline. However, the growth of *tetON-CAT1* cells decreased in the presence of doxycycline, reinforcing the view that elevated catalase levels reduce fitness in the absence of stress.

Catalase is a ferroprotein [34] expressed at relatively high basal levels in *C*. *albicans* (approximately 1.5 x 10^5^ molecules per cell [33]). In bacteria, catalase overexpression has been reported to affect the requirement for iron [50]. Therefore, we reasoned that the fitness defect conferred by high basal catalase levels in *C*. *albicans* might be mediated by an elevated cellular demand for iron. Hence, we tested whether iron supplementation can suppress this fitness defect. Growth of *tetON-CAT1* cells was measured in YPD containing doxycycline supplemented with different concentrations of ferric ions (Fig 9B). These data indicate that the fitness defect caused by ectopic catalase expression can be completely suppressed by iron supplementation. This suppression was due to the improved growth of doxycycline-treated *tetON-CAT1* cells in the presence of iron (S5A Fig). We also showed that iron supplementation suppresses the reduced fitness of doxycycline-treated *tetON-CAT1* cells in direct competition experiments with wild type (*CAT1*) cells (S5B Fig).

These observations suggested that high basal catalase expression increases the cellular demand for iron in *C*. *albicans*. To test this further we examined the impact of ectopic catalase expression upon key genes involved in iron assimilation and homeostasis: *CFL5* (encoding a ferric reductase that is induced in low iron), *FET3* (encoding a copper oxidase that is required for growth in low iron), *FRP1* (encoding a ferric reductase that is induced by iron chelation) and *FTR1* (encoding a high-affinity iron permease that is required for growth in low iron). All of these genes are targets of the iron-responsive transcriptional activator Sef1 [15]. *CFL5*, *FET3*, *FRP1* and *FTR1* transcript levels were measured relative to the *ACT1* mRNA internal control in *tetON-CAT1* cells grown in the presence and absence of doxycycline. Their levels were then normalised against those in doxycycline-treated wild type (*CAT1*) cells to exclude any potential effects of this treatment on these transcripts [51]. All four iron-responsive transcripts were strongly induced following *tetON-CAT1* induction (Fig 9C). Taken together, the data indicate that high basal catalase levels increase the requirement for iron in *C*. *albicans*.

## Discussion

This study has important implications for the impact of the key peroxide detoxifying enzyme, catalase, upon the stress resistance and virulence of the major fungal pathogen, *C*. *albicans*. Firstly, our analyses of new *cat1Δ* null mutants, in which potential *URA3* position effects have been circumvented [49], have reinforced the view that catalase is essential for normal levels of oxidative and combinatorial stress resistance in *C*. *albicans* (Figs 2 & 8). They also show that catalase contributes to the resistance of this pathogenic fungus against neutrophil killing (Fig 7). However, our most surprising finding was that, in contrast to the generally held view [23,40], catalase is not essential for the virulence of *C*. *albicans*, at least in models of disseminated candidiasis. This unexpected finding is supported by virulence assays in both short term and long term murine models of systemic infection, and in an accepted invertebrate model of systemic infection (Fig 6). This view is further reinforced by our *in vivo* competition experiments, in which the *cat1Δ* null mutant competed effectively against wild-type and catalase overexpressing strains for colonisation of the kidney, liver, spleen and brain (Fig 5). We suggest that the attenuated virulence of the *cat1Δ* mutants reported previously [23,40] might be explained by *URA3* position effects in these strains [49].

Why might catalase be important for oxidative stress resistance and yet apparently not required for systemic infection? The sensitivity of *cat1Δ* cells to neutrophil killing (Fig 7) does indicate that protection against peroxide is required in certain contexts *in vivo*. Therefore, this lack of *cat1Δ* virulence defect probably reflects the multifactorial nature of virulence phenotypes, as well as the nature of the systemic infection models often used to examine virulence. In these models sufficient fungal doses are applied to overcome immediate clearance by circulating phagocytes [47]. Furthermore, few of the fungal cells colonising the kidney appear to be exposed to oxidative stress [31].

Secondly, our data indicate that high *basal* levels of catalase promote the resistance of *C*. *albicans* to peroxide and combinatorial stress (Fig 2). These data reaffirm previous reports that elevated catalase expression promotes peroxide resistance [28,36]. Significantly, our data indicate that this phenotype is dependent on high basal levels of catalase at the point of stress imposition, rather than *CAT1* induction in response to stress. Three independent observations support this view. (A) *tetON-CAT1* cells are only protected against peroxide or combinatorial stress if these cells are pre-treated with doxycycline, not if doxycycline is only provided at the same time as the stress (Fig 2). (B) Clinical isolates that are relatively resistant to oxidative stress tend to express catalase at relatively high levels (Fig 3). (C) Unstressed *C*. *albicans* cell populations display heterogeneity in Cat1-GFP levels, and those cells that express more Cat1-GFP are less susceptible to killing by oxidative stress (Fig 4). Hydrogen peroxide is normally rapidly detoxified by wild-type *C*. *albicans* cells (within 60 minutes) in a catalase-dependent fashion [28]. Elevated basal levels of catalase presumably enhance cellular protection by accelerating the clearance of this reactive oxygen species. The heterogeneity in catalase expression within *C*. *albicans* populations, which might arise via stochastic differences between cells [52–54], appears to account, in large part, for the ability of a subset of *C*. *albicans* cells to survive an acute oxidative stress. This would appear to represent the first example in *C*. *albicans* of the kind of “bet-hedging” strategies that have been observed in bacterial and *S*. *cerevisiae* populations [55,56]. Furthermore, these observations are entirely consistent with the well-established observation that an adaptive response to a small dose of a particular stress can transiently endow yeasts with resistance to a subsequent acute dose of the same stress by inducing the expression of key stress protective functions. This observation has been reported for heat shock, osmotic and oxidative stress in *S*. *cerevisiae* for example [57,58], and has been extended to other yeasts including *C*. *albicans* [43,59,60].

Thirdly, our data provide key insights into the impact of catalase levels on the virulence of *C*. *albicans*. In our hands, direct competition assays suggested that elevated catalase levels might affect *C*. *albicans* colonisation of the kidney and brain (Fig 5). This is consistent with a parallel study which reported that catalase overexpression attenuates the virulence of *C*. *albicans* [36]. Roman and co-workers described this as “*a most unexpected result*” given that catalase overexpression enhances oxidative stress resistance. They speculate that this might have arisen via some alteration in fitness, which they were unable to detect *in vitro*, but which might interfere with activation of the Hog1 and Mpk1 MAP kinases [36]. In this study we show clearly in direct competition assays that elevated basal catalase levels attenuate the fitness of *C*. *albicans* in the absence of stress (Fig 8). We conclude that catalase overexpression confers a selective disadvantage in *C*. *albicans* in the absence of stress.

Fourthly, we have identified a major cause of this fitness defect. High basal catalase levels increase the cellular requirement for iron in *C*. *albicans*. We present two key observations that support this. (i) The fitness defect is suppressed by iron supplementation (Fig 9B and S4 Fig). This effect, which has also been observed in bacteria [50], is probably mediated by the depletion of intracellular iron through high level expression of an abundant heme-requiring enzyme. (ii) Ectopic catalase expression induces the expression of iron-responsive genes that play key roles in iron scavenging and homeostasis: e.g. *CFL5*, *FET3*, *FRP1* and *FTR1* (Fig 9C). Therefore, the demand for iron and catalase expression are intimately linked in *C*. *albicans*. Both modulate the accumulation of intracellular ROS. Iron stimulates *CAT1* expression in *C*. *albicans* [16,61]. This increase in catalase affects iron demand and homeostasis (Fig 9B & 9C) and also enhances the detoxification of hydrogen peroxide, thereby decreasing the production of highly toxic hydroxyl radicals via the iron-dependent Fenton reaction [11]. Parallels exist in *S*. *cerevisiae*, where heterogeneity in superoxide dismutase (*SOD1*) gene expression affects the fitness of individual cells in the presence of copper [62].

The impact of catalase levels on the requirement for iron is likely to have a profound effect on *C*. *albicans* pathogenicity because iron homeostasis is tightly regulated during infection [10,15] and efficient iron assimilation is essential for colonisation of iron limiting niches in the mammalian host [7]. It would appear significant, therefore, that we observed reduced colonisation for catalase overexpressing cells in the kidney and brain, but not in the iron-rich liver and spleen (Fig 5).

In conclusion, elevated basal catalase levels appear to be a double-edged sword whereby they protect *C*. *albicans* against oxidative and combinatorial stresses imposed by the host while increasing the pathogen’s demand for an essential, but limiting micronutrient in the host. This double-edged sword would appear to account for the apparently counterintuitive observation that catalase overexpression in *C*. *albicans* decreases host colonisation in some tissues [36]. It also helps to explain why *C*. *albicans* has not evolved to express the high levels of catalase that would protect it from phagocytic killing [28,36].

## Materials and methods

### Strains, growth conditions and treatments

The strains used in this study are listed in S1 Table. *C*. *albicans* was routinely grown at 30°C, 200 rpm in YPD (2% dextrose, 2% mycological peptone, 1% yeast extract) containing 20 μg/ml doxycycline (Dox) when required. On the day of an experiment, overnight cultures were diluted into fresh YPD to an OD_600_ of 0.2, and incubated at 30°C at 200 rpm until they reached an OD_600_ of 0.8, whereupon they were subjected to the appropriate treatment and analysed. Plates were incubated for 48 h at 30°C.

Osmotic stress was applied with 1 M NaCl and oxidative stress was applied with H_2_O_2_ at the specified concentration. Combinatorial stress was imposed using 1 M NaCl plus 5 mM H_2_O_2_ as described previously [28,63].

Robotic plating was performed using a Singer RoToR robot (Singer Instruments, Watchet, UK). Fitness was assayed by monitoring growth in microtitre plates at OD_600_ every 20 min for 48 h, and data from independent triplicate experiments were analysed.

### Strain construction

The *CAT1* locus was deleted from the *C*. *albicans* strain CEC2908 using the Clox system as previously described [64] (S2 Table), thereby generating the homozygous *cat1*Δ null mutant Ca2037 (S1 Table). Using published procedures [38], the *C*. *albicans CAT1* ORF was then cloned into barcoded CIp10-P_*TET*_-GTw plasmids and these plasmids were integrated at the *RPS1* locus in *C*. *albicans* Ca2037 (S1 Table) to generate the strains Ca2038, Ca2040, Ca2041, Ca2043, Ca2044, and Ca2046 (S1 Table). Empty barcoded CIp10-P_*TET*_-GTw plasmids were transformed into *C*. *albicans* CEC2908 to create strains Ca2084, Ca2085 and Ca2087 (S1 Table). Empty barcoded CIp10-P_*TET*_-GTw plasmids were also transformed into *C*. *albicans* Ca2037 to generate strains Ca2089, Ca2092 and Ca2130 (S1 Table). This created an isogenic set of nine barcoded wild-type (*CAT1*), null (*cat1*Δ) and *tetON-CAT1* strains. Their 25 bp barcodes are described in S3 Table.

The *CAT1-GFP/CAT1-GFP* strain Ca2213 (S1 Table) was constructed by PCR amplifying *CAT1-GFP-URA3* and *CAT1-GFP-HIS1* cassettes (S2 Table) [65] and integrating these sequentially at the 3’-end of the *CAT1* alleles in *C*. *albicans* RM1000 (S3 Table).

### Barcode sequencing

To quantify the relative concentration of each barcoded strain in mixed populations of *tetON* strains, genomic DNA was prepared from the populations by phenol: chloroform extraction method [66]. A 60 bp region carrying the barcodes (S3 Table) was amplified with common primers (S2 Table) using the KAPA HiFi HotStart ReadyMix PCR Kit (KAPA Biosystems, London, UK) and ethanol precipitated. These purified amplicons, which contained the Illumina overhang, were then indexed with Illumina Nextera XT v2 indices (Illumina, Inc., San Diego, CA, USA). Briefly, the dual indexed Illumina libraries were prepared with 5 μl of DNA, 5 μl each of i5 and i7 index primer, 25 μl KAPA HiFi HotStart ReadyMix, and 10 μl of PCR grade water and PCR amplified (95°C for 3 min; 8 cycles of 95°C for 30 sec, 55°C for 30 sec and 72°C for 30 sec; 72°C for 5 min; and a final hold at 4°C) on a Life Technologies Veriti thermal cycler (Thermo Fisher Scientific, Waltham, MA, USA). The libraries were purified and size selected using a double size selection with SPRIselect (Beckman Coulter, Brea, CA, USA) with a SPRIselect to sample ratio of 0.85x followed by 1.0x. Libraries were quantified using the Thermo Fisher Scientific Quant-iT dsDNA High Sensitivity Assay and the fluorescence measured on a BMG Labtech FLUOstar Omega microplate reader (BMG Labtech GmbH, Ortenberg, DE). The quality and size (bp) of the libraries were analysed on an Agilent 2200 TapeStation with High Sensitivity D1000 ScreenTapes (Agilent Technologies, Santa Clara, CA, USA). The libraries were pooled in equimolar amounts and sequenced on an Illumina MiSeq Sequencing System using MiSeq v3 chemistry with 76 bp paired-end reads. Base calling and fastq output files were generated with RTA v1.18.54 software on the MiSeq instrument.

To analyse the barseq data, a wrapper script was coded over the open source BBDuk tool (BBMap suite version 35.43 [67]). The wrapper visits each sample directory and runs the 3rd-party *bbduk*.*sh* script over each of the compressed read 1 and read 2 FASTQ files, generating corresponding FASTQ output files for the “matched” and “not-matched” reads for each barcode. The wrapper then computes the total number of reads for each barcode and its abundance relative to the total number of barcode reads. The barseq data are presented as the relative abundance of a barcode normalised to its starting concentration in the population. Means and standard deviations from three replicate measurements are presented.

### Transcript levels

RNA was extracted from *C*. *albicans* cells using the Zymo Research YesStar RNA Kit (Cambridge Bioscience, Cambridge, UK). cDNA was prepared using SuperScript II reverse transcriptase from Invitrogen (Fisher Scientific, Loughborough, UK), and qRT-PCR was performed with a Roche Light Cycler 480 II using the primers described in S2 Table. Transcript levels were measured in triplicate, expressed relative to the internal *ACT1* mRNA control [28], and then normalised against the levels in doxycycline-treated wild type (*CAT1*) cells to exclude potential effects of doxycycline on these transcripts.

### Catalase assays

*C*. *albicans* cells grown in YPD containing 0 or 20 μg/μl Dox were subjected to no stress or one hour of 5 mM H_2_O_2_, protein extracts prepared, and catalase activities measured using the BioAssay Systems EnzyChrom catalase assay kit (Universal Biologicals Ltd., Cambridge, UK), according to the manufacturer’s instructions [28]. Assays were performed in triplicate.

### Cat1-GFP expression

*CAT1-GFP* and *ACT1-GFP* expression in *C*. *albicans* cell populations was examined and cell subsets isolated using the BD Influx cell sorter. Heterogeneity in *C*. *albicans* cell size was first analysed (Forward Scatter (FSC), Side Scatter (SSC)) and cells of similar size selected (S2 & S3 Figs). Cells were then sorted on the basis of their GFP expression level (S2B Fig). Cells (n = 200) that expressed GFP at relatively low levels and 200 cells expressing GFP at high levels were plated onto YPD containing various concentrations of H_2_O_2_. These two populations sorted were separated to 99% purity. Control experiments were performed to confirm cell viability by propidium iodide staining (2 μg/ml). Data were analysed using BD FACS software and Flowjo software version 10.0.8.

*CAT1-GFP* cells were visualized using a DeltaVision Core microscope (Applied Precision, Issaquah, WA). Western blotting was performed as described previously [68].

### Cell viability and ROS accumulation

Cell viability was assayed by measuring colony forming units (CFU) on YPD plates and by propidium iodide (PI) staining and flow cytometry on a BD LSR II, as described previously [28,63].

Intracellular ROS accumulation was measured by staining the cells with 20 μM dihydroethidium for one hour in darkness, at 30°C and 200 rpm, and then analysed using a BD LSR II flow cytometer. Data were analysed using Flowjo software version 10.0.8.

### Neutrophil killing assays

Blood from healthy donors was obtained according to the protocol approved by the University of Aberdeen College Ethics Review Board (Application number—CERB/2012/11/676). Polymorphonuclear (PMN) cells, or neutrophils, were isolated from this blood using Histopaque-1119 and Histopaque-1077 (Sigma Aldrich) as described previously [28]. *C*. *albicans* cells pre-grown with 20 μg/ml Dox were incubated with PMNs (1:10 ratio of yeasts to neutrophils) for 2 h in RPMI 1640 containing 10% heat inactivated foetal bovine serum. After incubation the PMNs were treated with 0.25% sodium docecyl sulphate and DNase I and yeast survival determined by assaying CFU. Data from eight healthy donors are presented with their means and standard deviation.

### Virulence assays

The virulence of *C*. *albicans* wild type and *cat1*Δ cells were measured in a short term murine model of systemic candidiasis [47]. Strains were pre-grown in YPD and injected intravenously (4 x 10^4^ CFU/g body weight) into the lateral tail vein of 6–10 week old female BALB/c mice (Envigo, UK). Mice were randomly assigned to cages (n = 6 per group) and inocula were randomly assigned to cages. Infections were allowed to proceed for 4 days whereupon the mice were humanely culled by cervical dislocation and fungal burdens (CFU/g) determined in the kidneys. Fungal burden and weight loss were used as measures of virulence [47].

The virulence of *C*. *albicans* wild type and *cat1*Δ strains were also tested in a longer term mouse infection model. Again, *C*. *albicans* cells were injected into the tail veins of 6–10 week old female BALB/c mice (3 x 10^4^ CFU/g body weight). Once again, the mice were randomly assigned to cages (n = 8 per group) and inocula were assigned randomly to cages. The mice were monitored and weighed daily, and were humanely culled when they had lost 20% of their body weight and death recorded as having occurred on the following day. Experiments were continued for a maximum of 14 days, when all surviving mice were culled and analysed. The data are presented as Kaplan-Meier survival curves (log rank tests).

To directly compare the colonisation of *C*. *albicans tetON* strains in the mouse model of systemic candidiasis, the strains were pre-grown in YPD containing 0 or 20 μg/ml doxycycline and injected into the tail vein of 6–10 week old female BALB/c mice (4 x10^4^ CFU/g body weight: n = 6 mice per group). Mice were gavaged with 100 μl of 0 or 40 mg/ml doxycycline. Infections were allowed to proceed for up to 4 days. Mice were culled, their kidneys, spleen, liver and brain removed and homogenized in 500 μl saline, and the entire sample from each organ plated onto YPD. The fungal colonies from each individual organ were then pooled, and genomic DNA prepared for barseq (above).

The virulence of *C*. *albicans* strains was also evaluated using the invertebrate *Galleria mellonella* infection model [69]. For each *C*. *albicans* strain, 10^5^ cells were injected into 20 *Galleria* larvae (6^th^ instar: BioSystems Technology, Exeter, UK). Sterile PBS was injected into control larvae. Survival was monitored for 5 days at 37°C, represented using Kaplan-Meier curves, and analysed using log rank tests.

### Ethics statement

All animal experiments were conducted in compliance with United Kingdom Home Office licenses for research on animals, and were approved by the University of Aberdeen Ethical Review Committee (project license number PPL 70/8583). Animal experiments were minimised, and all animal experimentation was performed using approaches that minimised animal suffering and maximised our concordance with EU Directive 2010/63/EU.

Power analyses based on data generated in previous experiments were applied to estimate the minimum number of animals per group required to achieve statistically robust differences (P <0.05). The power analyses to determine group size for the short term systemic infection model were based on the variation in fungal burdens between animals, whereas those for the long term model were based upon mean survival times. Animals were monitored at least twice daily for signs of distress, which was minimised by expert handling. Euthanasia was performed humanely by cervical dislocation when animals showed signs of progressive illness (e.g. ruffled coat, hunched posture, unwillingness to move and 20% loss of initial body weight). During these studies there were no unexpected deaths. Analgesia and anaesthesia were not required in this study.

### Statistical analysis

Statistical analyses were performed in GraphPad Prism 5 and IBM SPSS Statistics (v24.0.0). Two tailed Mann-Whitney U analysis was used to test the statistical difference between two sets of data with a non-parametric distribution. Associations between growth parameters, such as doubling time, lag phase or propidium iodide staining, were determined by one-way and two-way ANOVA and Dunnett post-hoc t-tests. Unstressed samples were used as controls and the values of other samples were compared against these controls. The following *p*-values were considered: * *p <* 0.05; ** *p* <0.01; *** *p* < 0.001; **** *p* < 0.0001.

## Supporting information

S1 Fig Stress resistance of *C*. *albicans tetON-CAT1* and *ACT1-CAT1* strains. *C*. *albicans* cultures were pre-grown in YPD with 20 μM doxycycline, dilutions spotted onto YPD plates containing H_2_O_2_ and/or NaCl stresses at the specified concentrations, and photographed after 24 h growth at 30°C: *CAT1/CAT1*, Ca674; *CAT1/cat1*Δ, Ca1862; *cat1*Δ*/cat1*Δ, Ca1864; *ACT1*_*p*_*-CAT1*, Ca2031; *cat1*Δ *tetON-CAT1*, Ca2038; *CAT1 tetON-empty*, Ca2084; *cat1*Δ *tetON-empty*, Ca2089 (S1 Table).
(PDF)

S2 Fig Gating strategy for the analysis of *C*. *albicans* Cat1-GFP expressing cells. Exponential populations of *C*. *albicans CAT1-GFP* cells (Ca2213: S1 Table) growing in YPD at 30°C were subjected to fluorescence activated cell sorting. **(A)** First, singlets were selected and doublets excluded by analysing the FSC signals height *versus* area. **(B)** Next, cells of similar size were selected by analysing the FSC *versus* SSC. **(C)** This cell population was analysed for their GFP fluorescence intensity (530–540 nm) by plotting GFP against a dump channel (610-620nm). *CAT1-GFP* cells were compared with control cells with no GFP (Ca674). *CAT1-GFP* cells with relatively low levels of Cat1-GFP (pink), and cells with relatively high Cat1-GFP levels (cyan) were sorted using the single cell modus of a BD Influx sorter. **(D)** FACS sorting of *C*. *albicans* cells expressing relatively low or high levels of Cat1-GFP in the absence of stress (same figure as Fig 4D). **(E)** These FACS sorted cells (n = 200 per group) were plated onto YPD containing different concentrations of H_2_O_2_, and percentage survival (CFUs) calculated relative to the no stress control (same figure as Fig 4E). Means and standard deviations from three replicates are presented: *, *p* ≤ 0.05; **, *p* ≤ 0.01; ***, *p* ≤ 0.001; ****, *p* ≤ 0.0001.
(PDF)

S3 Fig Analysis of *C*. *albicans ACT1*-*GFP* expressing cells. Exponential *C*. *albicans ACT1-GFP* cells (Ca230: S1 Table) were grown in the same way as for S2 Fig, and then subjected to fluorescence activated cell sorting, as before. **(A)** Singlets were selected and doublets excluded by analysing the FSC signals height *versus* area. **(B)** Cells of similar size were then selected by analysing the FSC *versus* SSC. **(C)** These cells were analysed for their GFP fluorescence intensity as described in S2 Fig. *ACT1-GFP* cells with relatively low (pink) and high GFP levels (cyan) were sorted using the single cell modus of a BD Influx sorter. **(D)** FACS sorting of *C*. *albicans* cells expressing relatively low or high *ACT1*-*GFP* levels in the absence of stress. **(E)** These FACS sorted cells (n = 200 per group) were plated onto YPD containing different concentrations of H_2_O_2_, and percentage survival (CFUs) calculated relative to the no stress control.
(PDF)

S4 Fig Loss of phenotype in some *tetON-CAT1* isolates. *TetON-CAT1* isolates 1, 4 and 10 (Ca2038, Ca2041, Ca2044: S1 Table) behaved differently *in vivo*: isolate 1 displayed decreased colonisation in certain tissues (Fig 5), whereas isolates 4 and 10 did not (see text). Therefore, we tested whether isolates 4 and 10 had lost their phenotype over time. To achieve this we compared the “old” isolates 1, 4 and 10 (Ca2038, Ca2041, Ca2044) with “new” isolates (Ca2040, Ca2043, Ca2046). (Throughout this study, all experiments were performed on strains that had been freshly plated from frozen -80°C stocks. “Old” isolates were taken routinely from the same -80°C tubes over a twelve month period. These stocks were not thawed: small volumes were chipped from their frozen surface and plated. Nevertheless, the temperature of these tubes must have increased transiently from -80°C at regular intervals over this period. In contrast, “new” isolates were from identical -80°C stocks that were not touched over this period.) **(A)** As described in Fig 1, catalase activities were measured in *C*. *albicans* cells grown in YPD containing 0 or 20 μM doxycycline (- or + Dox, respectively): *cat1*Δ, Ca2089; wild-type, WT, Ca2084; blue, old *tetON-CAT1* isolates, Ca2038, Ca2041, Ca2044; red, new *tetON-CAT1* isolates, Ca2040, Ca2043, Ca2046 (S1 Table). Wild-type and *cat1*Δ cultures were exposed to 0 or 5 mM H_2_O_2_ for one hour before analysis. Means and standard deviations from three independent replicate experiments are shown, and the data were analysed using one-way ANOVA with Tukey’s post-hoc test: *, *p* ≤ 0.05; **, *p* ≤ 0.01; ***, *p* ≤ 0.001; ****, *p* ≤ 0.0001. **(B)** The fitness of these old and new *tetON-CAT1* strains was compared *in vitro* by examining their growth (biomass formation; final OD_600_) on YPD containing or lacking doxycycline (no stress). All of the isolates displayed similar growth in the absence of doxycycline, and the wild-type (*CAT1*) controls remained unaffected by doxycycline. The presence of doxycycline led to decreased growth for all of the new *C*. *albicans tetON-CAT1* strains and for the old *tetON-CAT1-*1 strain (Fig S4B). No significant effect of doxycycline on growth and fitness was observed for the old *C*. *albicans tetON-CAT1* isolates 4 and 10. This correlated with a reduction in catalase levels in these isolates over time (Fig S4A). These data indicate that the old isolates 4 and 10 had indeed lost their *in vitro* fitness defect over time, thereby explaining their lack of phenotype *in vivo*.
(PDF)

S5 Fig Iron suppresses the fitness defect of doxycycline-treated *tetON-CAT1 C*. *albicans* cells. **(A)** Iron supplementation restores the growth of doxycycline-treated *tetON-CAT1 C*. *albicans* cells to normal, while reducing the growth of wild type and *cat1*Δ null cells. The growth of new *C*. *albicans* isolates was monitored (OD_600_) in YPD containing 0 or 20 μM doxycycline plus different concentrations of FeCl_3_: black circles, wild type *CAT1-*21 (Ca2084) cells plus doxycycline; red circles, null *cat1*Δ-28 (Ca2089) cells plus doxycycline; pale open circles, *tetON-CAT1-*1 (Ca2040) cells with no doxycycline; blue circles, *tetON-CAT1-*1 (Ca2040) cells with plus doxycycline (S1 Table). **(B)** Iron supplementation restores the ability of doxycycline-treated *tetON-CAT1* cells to compete with wild type *C*. *albicans* cells in mixed cultures. Wild type (Ca2084) and *tetON-CAT1-*1 (Ca2040) cells were mixed in roughly equal proportions and grown for 12 h in YPD containing 20 μM doxycycline plus 0 or 500 μM FeCl_3_. The proportion of each cell type at the end of these competition experiments (% colonies) was determined by replica plating single colonies onto YPD plates lacking or containing combinatorial stress (1 M NaCl plus 5 mM H_2_O_2_): wild type cells are sensitive whilst *tetON-CAT1* cells resistant to this stress. Means and standard deviations from three replicates are presented: *, *p* ≤ 0.05; **, *p* ≤ 0.01.
(PDF)

S1 Table Strains used in this study. (PDF)

S2 Table Primers used in this study. (PDF)

S3 Table Barcodes used in this study. (PDF)

## References

[1] Hawksworth DL. Global species numbers of fungi: are tropical studies and molecular approaches contributing to a more robust estimate? Biodivers. Conserv. 2012 21:2425–2433.

[2] de Hoog GS, Guarro J, Gene J, Figueras MJ. Atlas of Clinical Fungi (2nd Edition) Centraalbureau voor Schimmelcultures, Utrecht, The Netherlands/Universitat Rovira i Virgili, Reus, Spain 2000. ISBN 90-70351-43-9.

[3] Brown GD, Denning DW, Gow NAR, Levitz SM, Netea MG, White TC. Hidden killers: human fungal infections. Science Trans. Med. 2012 4:165rv13. 10.1126/scitranslmed.3004404 23253612

[4] Nikolaou E, Agrafioti I, Stumpf M, Quinn J, Stansfield I, Brown AJ. Phylogenetic diversity of stress signalling pathways in fungi. BMC Evol. Biol. 2009 9:44 10.1186/1471-2148-9-44 19232129 PMC2666651

[5] Miramon P, Dunker C, Windecker H, Bohovych IM, Brown AJP, Kurzai O, Hube B. Cellular responses of *Candida albicans* to phagocytosis and the extracellular activities of neutrophils are critical to counteract carbohydrate starvation, oxidative and nitrosative stress. PLoS ONE. 2012 7(12): e52850 10.1371/journal.pone.0052850 23285201 PMC3528649

[6] Brown AJP, Cowen LE, Di Pietro A, Quinn J. Stress adaptation In “*The Fungal Kingdom*” [Heitman J, Howlett B, Crous P, Stukenbrock E, James T, Gow NAR, eds.], ASM Press, 2016. (in press).

[7] Ramanan N, Wang Y. A high-affinity iron permease essential for *Candida albicans* virulence. Science. 2000 288:1062–1064. 10807578 10.1126/science.288.5468.1062

[8] Schrettl M, Bignell E, Kragl C, Joechl C, Rogers T, Arst HN Jr., Haynes K, Haas H. Siderophore biosynthesis but not reductive iron assimilation is essential for *Aspergillus fumigatus* virulence. J Exp. Med. 2004 200:1213–1219. 10.1084/jem.20041242 15504822 PMC2211866

[9] Jung WH, Hu G, Kuo W, Kronstad JW. Role of ferroxidases in iron uptake and virulence of *Cryptococcus neoformans*. Eukaryotic Cell. 2009 8:1511–1520. 10.1128/EC.00166-09 19700638 PMC2756870

[10] Potrykus J, Stead D, MacCallum DM, Urgast DS, Raab A, van Rooijen N, Feldmann J, Brown AJP. Fungal iron availability during deep seated candidiasis defined by a complex interplay of systemic and local events. PLoS Pathogens. 2013 9:e1003676 10.1371/journal.ppat.1003676 24146619 PMC3798425

[11] Stohs SJ, Bagchi D. Oxidative mechanisms in the toxicity of metal ions. Free Rad. Biol. Med. 1995 18:321–336. 7744317 10.1016/0891-5849(94)00159-h

[12] Potrykus J, Ballou ER, Childers DS, Brown AJP. Conflicting interests in the pathogen-host tug of war: Fungal micronutrient scavenging versus mammalian nutritional immunity. PLoS Pathogens. 2014 10: e1003910 10.1371/journal.ppat.1003910 24626223 PMC3953404

[13] Jung WH, Kronstad JW. Iron and fungal pathogenesis: a case study with *Cryptococcus neoformans*. Cell. Micro. 2008 10:277–284. 10.1111/j.1462-5822.2007.01077.x 18042257

[14] Almeida RS, Wilson D, Hube B. *Candida albicans* iron acquisition within the host. FEMS Yeast Res. 2009 9: 1000–1012. 10.1111/j.1567-1364.2009.00570.x 19788558

[15] Chen C, Pande K, French SD, Tuch BB, Noble SM. An iron homeostasis regulatory circuit with reciprocal roles in *Candida albicans* commensalism and pathogenesis. Cell Host Microbe. 2011 10:118–135. 10.1016/j.chom.2011.07.005 21843869 PMC3165008

[16] Singh RP, Prasad HK, Sinha I, Agarwal N, Natarajan K. Cap2-HAP complex is a critical transcriptional regulator that has dual but contrasting roles in regulation of iron homeostasis in *Candida albicans*. J. Biol. Chem. 2011 286:25154–25170. 10.1074/jbc.M111.233569 21592964 PMC3137088

[17] Haas H. Iron—a key nexus in the virulence of *Aspergillus fumigatus*. Front. Microbiol. 2012 3:28 10.3389/fmicb.2012.00028 22347220 PMC3272694

[18] Steen BR, Zuyderduyn S, Toffaletti DL, Marra M, Jones SJM, Perfect JR, Kronstad J. *Cryptococcus neoformans* gene expression during experimental cryptococcal meningitis. Eukaryotic Cell. 2003 2:1336–1349. 10.1128/EC.2.6.1336-1349.2003 14665467 PMC326655

[19] Lorenz MC, Bender JA, Fink GR. Transcriptional response of *Candida albicans* upon internalization by macrophages. Eukaryotic Cell. 2004 3:1076–1087. 10.1128/EC.3.5.1076-1087.2004 15470236 PMC522606

[20] Fan W, Kraus PR, Boily MJ, Heitman J. *Cryptococcus neoformans* gene expression during murine macrophage infection. Eukaryotic Cell. 2005 4:1420–1433. 10.1128/EC.4.8.1420-1433.2005 16087747 PMC1214536

[21] McDonagh A, Fedorova ND, Crabtree J, Yu Y, Kim S, et al Sub-telomere directed gene expression during initiation of invasive aspergillosis. PLoS Pathogens. 2008 4:e1000154 10.1371/journal.ppat.1000154 18787699 PMC2526178

[22] Wilson D, Thewes S, Zakikhany K, Fradin C, Albrech A, Almeida R, Brunke S, Grosse K, Martin R, Mayer F, Leonhardt I, Schild L, Seider K, Skibbe M, Slesiona S, Waechtler B, Jacobsen I, Hube B. Identifying infection-associated genes of *Candida albicans* in the postgenomic era. FEMS Yeast Res. 2009 9:688–700. 10.1111/j.1567-1364.2009.00524.x 19473261

[23] Wysong DR, Christin L, Sugar AM, Robbins PW, Diamond RD. Cloning and sequencing of a *Candida albicans* catalase gene and effects of disruption of this gene. Infect. Immun. 1998 66:1953–1961. 9573075 10.1128/iai.66.5.1953-1961.1998 PMC108149

[24] Hwang CS, Rhie GE, Oh JH, Huh WK, Yim HS, Kang SO. Copper- and zinc-containing superoxide dismutase (Cu/ZnSOD) is required for the protection of *Candida albicans* against oxidative stresses and the expression of its full virulence. Microbiology 2002 148:3705–3713 10.1099/00221287-148-11-3705 12427960

[25] Frohner IE, Bourgeois C, Yatsyk K, Majer O, Kuchler K. *Candida albicans* cell surface superoxide dismutases degrade host-derived reactive oxygen species to escape innate immune surveillance. Molec. Microbiol. 2008 71:240–252. 19019164 10.1111/j.1365-2958.2008.06528.x PMC2713856

[26] Patterson MJ, McKenzie CG, Smith DA, da Silva Dantas A, Sherston S, Veal EA, Morgan BA, MacCallum DM, Erwig LP, Quinn J. Ybp1 and Gpx3 signaling in *Candida albicans* govern hydrogen peroxide-induced oxidation of the Cap1 transcription factor and macrophage escape. Antiox. Redox Sig. 2013 19:2244–2260. 10.1089/ars.2013.5199 PMC3869436 23706023

[27] Phillips AJ, Sudbery I, Ramsdale M. Apoptosis induced by environmental stresses and amphotericin B in *Candida albicans*. Proc. Natl. Acad. Sci. USA. 2003 100:14327–14332. 10.1073/pnas.2332326100 14623979 PMC283591

[28] Kaloriti D, Jacobsen M, Yin Z, Patterson M, Tillmann A, Smith DA, Cook E, You T, Grimm MJ, Bohovych I, Grebogi C, Segal BH, Gow NAR, Haynes K, Quinn J, Brown AJP. Mechanisms underlying the exquisite sensitivity of *Candida albicans* to combinatorial cationic and oxidative stress that enhances the potent fungicidal activity of phagocytes. mBio. 2014 5:e01334–14. 10.1128/mBio.01334-14 25028425 PMC4161263

[29] Kos I, Patterson MJ, Znaidi S, Kaloriti D, da Silva Dantas A, Herrero-de-Dios CM, d'Enfert C, Brown AJP, Quinn J. Mechanisms underlying the delayed activation of the Cap1 transcription factor in *Candida albicans* following combinatorial oxidative and cationic stress are important for phagocytic potency. mBio. 2016 7:e00331–16. 10.1128/mBio.00331-16 27025253 PMC4817257

[30] Enjalbert B, Smith DA, Cornell MJ, Alam I, Nicholls S, Brown AJP, Quinn J. Role of the Hog1 stress-activated protein kinase in the global transcriptional response to stress in the fungal pathogen *Candida albicans*. Molec. Biol. Cell. 2006 17:1018–1032. 10.1091/mbc.E05-06-0501 16339080 PMC1356608

[31] Enjalbert B, MacCallum D, Odds FC, Brown AJP. Niche-specific activation of the oxidative stress response by the pathogenic fungus *Candida albicans*. Infect. Immun. 2007 75:2143–2151. 10.1128/IAI.01680-06 17339352 PMC1865731

[32] Brown AJP, Haynes K, Gow NAR, Quinn J. Stress responses in *Candida* In *Candida and Candidiasis* 2nd Edition (Clancy CJ, Calderone RA, eds.) ASM Press, 2012 pp225–242.

[33] Komalapriya C, Kaloriti D, Tillmann AT, Yin Z, Herrero-de-Dios C, Jacobsen MD, Belmonte RC, Cameron G, Haynes K, Grebogi C, de Moura APS, Gow NAR, Thiel M, Quinn J, Brown AJP, Romano MC. Integrative model of oxidative stress adaptation in the fungal pathogen *Candida albicans*. PLoS ONE. 2015 10: e0137750 10.1371/journal.pone.0137750 26368573 PMC4569071

[34] Mate MJ, Zamocky M, Nykyri LM, Herzog C, Alzari PM, Betzel C, Koller F, Fita I. Structure of catalase-A from *Saccharomyces cerevisiae*. J. Molec. Biol. 1999 268:135–149. 10.1006/jmbi.1998.2453 9931255

[35] Zamocky M, Gasselhuber B, Furtmuller PG, Obinger C. Turning points in the evolution of peroxidase—catalase superfamily: molecular phylogeny of hybrid heme peroxidases. Cell. Molec. Life Sci. 2014 71:4681–4696. 10.1007/s00018-014-1643-y 24846396 PMC4232752

[36] Roman E, Prieto D, Martin R, Correia I, Arango ACM, Alonso-Monge R, Zaragoza O, Pla J. Role of catalase overproduction in drug resistance and virulence in *Candida albicans*. Future Microbiol. 2016 10.2217/fmb-2016-0067 27690640

[37] Chauvel M, Nesseir A, Cabral V, Znaidi S, Goyard S, et al A versatile overexpression strategy in the pathogenic yeast *Candida albicans*: identification of regulators of morphogenesis and fitness. PLoS ONE. 2012 7(9):e45912 10.1371/journal.pone.0045912 23049891 PMC3457969

[38] Cabral V, Chauvel M, Firon A, Legrand M, Nesseir A, et al Modular gene over-expression strategies for *Candida albicans*. Methods Molec. Biol. 2012 845:227–244. 22328378 10.1007/978-1-61779-539-8_15

[39] Cabral V, Znaidi S, Walker LA, Martin-Yken H, Dague E, et al targeted changes of the cell wall proteome influence *Candida albicans* ability to form single- and multi-strain biofilms. PLoS Pathogens. 2014 10(12):e1004542 10.1371/journal.ppat.1004542 25502890 PMC4263760

[40] Nakagawa Y, Kanbe T, Mizuguchi I. Disruption of the human pathogenic yeast *Candida albicans* catalase gene decreases survival in mouse-model infection and elevates susceptibility to higher temperature and to detergents. Microbiol. Immunol. 2003 47:395–403. 12906099 10.1111/j.1348-0421.2003.tb03376.x

[41] Odds FC, Bougnoux ME, Shaw DJ, Bain JM, Davidson AD, Diogo D, Jacobsen MD, Lecomte M, Li SY, Tavanti A, Maiden MCJ, Gow NAR, d'Enfert C. Molecular phylogenetics of *Candida albicans*. Eukaryotic Cell. 2007 6:1041–1052. 10.1128/EC.00041-07 17416899 PMC1951527

[42] Mallick EM, Bergeron AC, Jones SK, Newman ZR, Brothers KM, Creton R, Wheeler RT, Bennett RJ. Phenotypic plasticity regulates *Candida albicans* interactions and virulence in the vertebrate host. Front. Microbiol. 7:780 10.3389/fmicb.2016.00780 27303374 PMC4880793

[43] Smith DA, Nicholls S, Morgan BA, Brown AJP, Quinn J. A Conserved Stress-Activated Protein Kinase Regulates a Core Stress Response in the Human Pathogen *Candida albicans*. Molec. Biol. Cell. 2004 15:4179–4190. 10.1091/mbc.E04-03-0181 15229284 PMC515350

[44] Strijbis K. Compartmentalization of metabolic pathways in Candida albicans: a matter of transport. PhD thesis, University of Amsterdam. 2009. uvapub:64396

[45] Travisano M, Velicer GJ. Strategies of microbial cheater control. Trends Microbiol. 2004 12:72–78. 10.1016/j.tim.2003.12.009 15036323

[46] MacLean RC, Gudelj I. Resource competition and social conflict in experimental populations of yeast. Nature. 2006 441:498–501. 10.1038/nature04624 16724064

[47] MacCallum DM, Coste A, Ischer F, Jacobsen MD, Odds FC, Sanglard D. Genetic dissection of azole resistance mechanisms in *Candida albicans* and their validation in a mouse model of disseminated infection. Antimicrob. Agents Chemother. 2010 54:1476–1483. 10.1128/AAC.01645-09 20086148 PMC2849354

[48] Murad AMA, Lee PR, Broadbent ID, Barelle CJ, Brown AJP. CIp10, an efficient and convenient integrating vector for *Candida albicans*. Yeast. 2000 16:325–327 10.1002/1097-0061(20000315)16:4<325::AID-YEA538>3.0.CO;2-# 10669870

[49] Brand A, MacCallum DM, Brown AJP, Gow NAR, Odds FC. Ectopic expression of *URA3* can influence the virulence phenotypes and proteome of *Candida albicans* but can be overcome by targeted reintegration of *URA3* at the *RPS10* locus. Eukaryotic Cell. 2004 3:900–909 10.1128/EC.3.4.900-909.2004 15302823 PMC500875

[50] Faulkner MJ, Ma Z, Fuangthong M, Helmann JD. Derepression of the *Bacillus subtilis* PerR peroxide stress response leads to iron deficiency. J. Bacteriol. 2012 194:1226–1235. 10.1128/JB.06566-11 22194458 PMC3294777

[51] Fioria A, Van Dijck P. Potent synergistic effect of doxycycline with fluconazole against *Candida albicans* is mediated by interference with iron homeostasis. Antimicrob. Agents Chemother. 2012 56:3785–3796. 10.1128/AAC.06017-11 22564841 PMC3393430

[52] Elowitz MB, Levine AJ, Siggia ED, Swain PS. Stochastic gene expression in a single cell. Science. 2002 297:1183–1186. 10.1126/science.1070919 12183631

[53] Sumner ER, Avery AM, Houghton JE, Robins RA, Avery SV. Cell cycle- and age-dependent activation of Sod1p drives the formation of stress resistant cell subpopulations within clonal yeast cultures. Molec. Microbiol. 2003 50:857–870. 14617147 10.1046/j.1365-2958.2003.03715.x

[54] Pedraza JM, van Oudenaarden A. Noise propagation in gene networks. Science. 2005 307:1965–1969. 10.1126/science.1109090 15790857

[55] Thattai M, van Oudenaarden A. Stochastic gene expression in fluctuating environments. Genetics. 2004 167: 523–530. 15166174 10.1534/genetics.167.1.523 PMC1470854

[56] Levy SF, Ziv N, Siegal ML. Bet hedging in yeast by heterogeneous, age-correlated expression of a stress protectant. PLoS Biol. 2012 10: e1001325 10.1371/journal.pbio.1001325 22589700 PMC3348152

[57] Lindquist S. The heat-shock response. Annu Rev Biochem. 1986 55:1151–1191. 10.1146/annurev.bi.55.070186.005443 2427013

[58] Berry DB, Gasch AP. Stress-activated genomic expression changes serve a preparative role for impending stress in yeast. 2008 Molec. Biol. Cell. 19:4580–4587. 10.1091/mbc.E07-07-0680 18753408 PMC2575158

[59] Enjalbert B, Nantel A, Whiteway M. Stress-induced gene expression in *Candida albicans*: absence of a general stress response. 2003 Molec. Biol. Cell. 14:1460–1467. 10.1091/mbc.E02-08-0546 12686601 PMC153114

[60] Komalapriya C, Kaloriti D, Tillmann AT, Yin Z, Herrero-de-Dios C, Jacobsen MD, Belmonte RC, Cameron G, Haynes K, Grebogi C, de Moura APS, Gow NAR, Thiel M, Quinn J, Brown AJP, Romano MC. Integrative model of oxidative stress adaptation in the fungal pathogen *Candida albicans*. 2015 PLoS ONE. 10:e0137750 10.1371/journal.pone.0137750 26368573 PMC4569071

[61] Lan CU, Rodarte G, Murillo LA, Jones T, Davis RW, Dungan J, Newport G, Agabian N. Regulatory networks affected by iron availability in *Candida albicans*. Molec. Microbiol. 2004 53:1451–1469. 15387822 10.1111/j.1365-2958.2004.04214.x

[62] Avery SV. Phenotypic diversity and fungal fitness. Mycologist. 2005 19:74–80.

[63] Kaloriti D, Tillmann A, Cook E, Jacobsen M, You T, Lenardon M, Ames L, Barahona M, Chandrasekaran K, Coghill G, Goodman D, Gow NAR, Grebogi C, Ho HL, Ingram P, McDonagh A, de Moura AP, Pang W, Puttnam M, Radmaneshfar E, Romano MC, Silk D, Stark J, Stumpf M, Thiel M, Thorne T, Usher J, Yin Z, Haynes K, Brown AJP. Combinatorial stresses kill pathogenic *Candida* species. Med. Mycol. 2012 50:699–709. 10.3109/13693786.2012.672770 22463109 PMC3483063

[64] Shahana S, Childers DS, Ballou ER, Bohovych I, Odds FC, Gow NAR, Brown AJP. New *Clox* systems for rapid and efficient gene disruption in *Candida albicans*. PLoS ONE. 2014 9:e100390 10.1371/journal.pone.0100390 24940603 PMC4062495

[65] Gerami-Nejad M, Berman J, Gale CA. Cassettes for PCR-mediated construction of green, yellow, and cyan fluorescent protein fusions in *Candida albicans*. Yeast. 2001 18:859–864. 10.1002/yea.738 11427968

[66] Hoffman CS, Winston F. A ten minute DNA preparation from yeast efficiently releases autonomous plasmids for transformation of *Escherichia coli*. Gene. 1987 57:267–272. 3319781 10.1016/0378-1119(87)90131-4

[67] Bushnell, B. BBMap (Version 35.43). 2015. Retrieved from https://sourceforge.net/projects/bbmap/.

[68] Leach MD, Stead DA, Argo E, MacCallum DM, Brown AJP. Proteomic and molecular analyses highlight the importance of ubiquitination for stress resistance, metabolic adaptation, morphogenetic regulation and virulence of *Candida albicans*. Molec. Microbiol. 2011 79:1574–1593. 21269335 10.1111/j.1365-2958.2011.07542.x PMC3084552

[69] Fallon J, Kelly J, Kavanagh K. *Galleria mellonella* as a model for fungal pathogenicity testing. Methods Molec. Biol. 2012 845:469–485. 22328396 10.1007/978-1-61779-539-8_33

