# Supplementary figures and images for: Elevated catalase expression in a fungal pathogen is a double-edged sword of iron
Source: PLoS Pathog. 2017 May 22;13(5):e1006405. doi: 10.1371/journal.ppat.1006405 (PMC5456399; doi:10.1371/journal.ppat.1006405)

Supplementary Figure 1

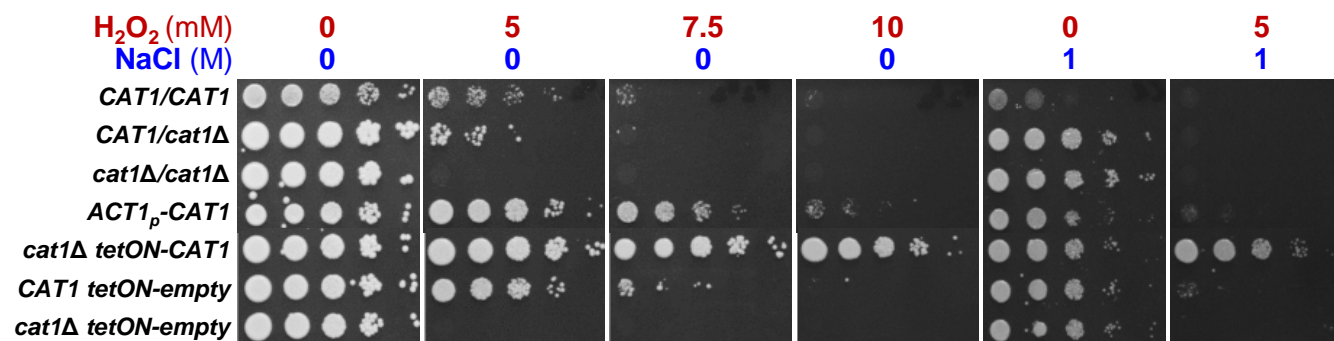

Supplement: S1 Fig — C. albicans cultures were pre-grown in YPD with 20 μM doxycycline, dilutions spotted onto YPD plates containing H2O2 and/or NaCl stresses at the specified concentrations, and photographed after 24 h growth at 30°C: CAT1/CAT1, Ca674; CAT1/cat1Δ, Ca1862; cat1Δ/cat1Δ, Ca1864; ACT1p-CAT1, Ca2031; cat1Δ tetON-CAT1, Ca2038; CAT1 tetON-empty, Ca2084; cat1Δ tetON-empty, Ca2089 (S1 Table). (PDF) [file ppat.1006405.s001.pdf]

Supplementary Figure 2

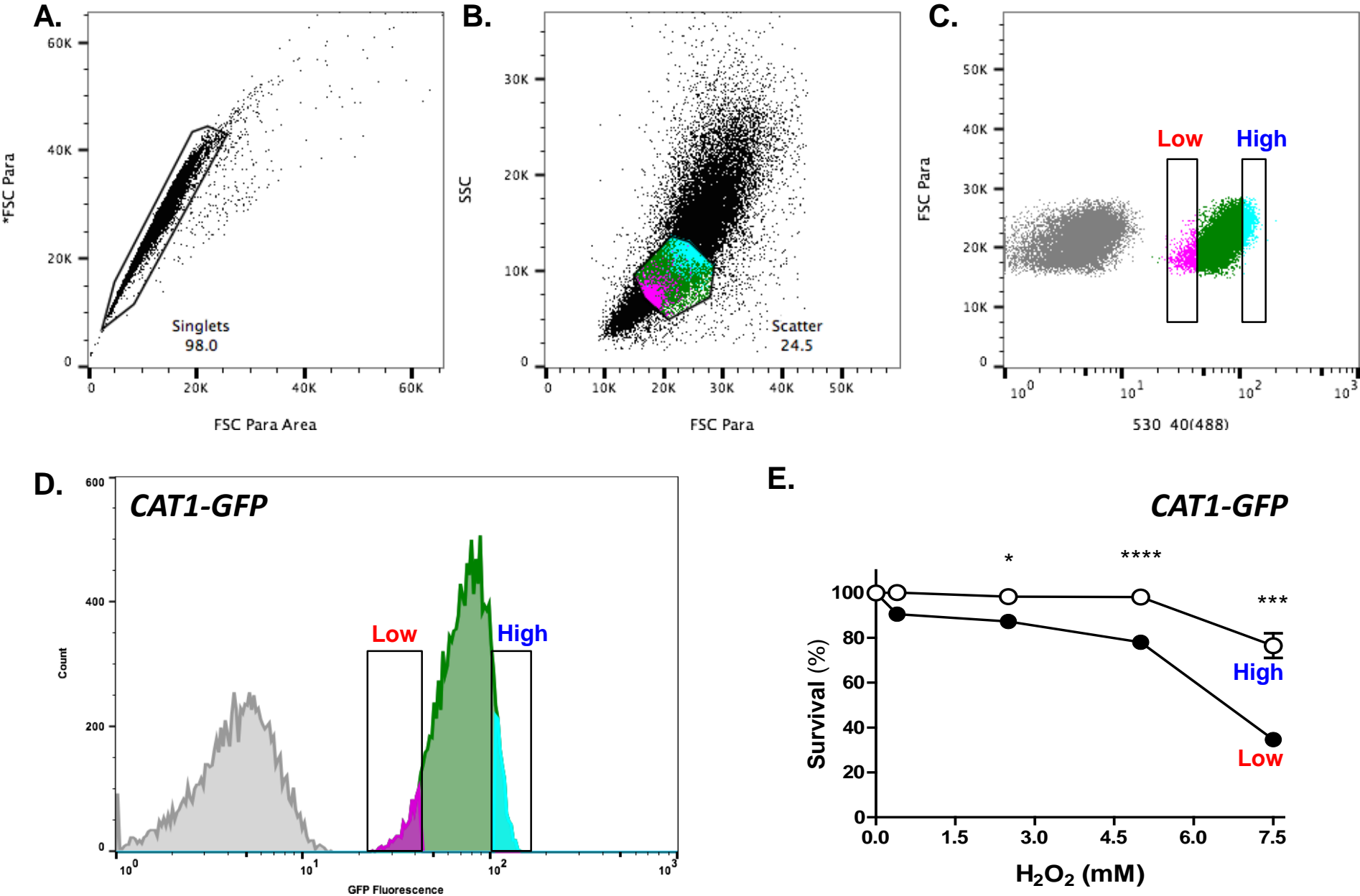

Supplement: S2 Fig — Exponential populations of C. albicans CAT1-GFP cells (Ca2213: S1 Table) growing in YPD at 30°C were subjected to fluorescence activated cell sorting. (A) First, singlets were selected and doublets excluded by analysing the FSC signals height versus area. (B) Next, cells of similar size were selected by analysing the FSC versus SSC. (C) This cell population was analysed for their GFP fluorescence intensity (530–540 nm) by plotting GFP against a dump channel (610-620nm). CAT1-GFP cells were compared with control cells with no GFP (Ca674). CAT1-GFP cells with relatively low levels of Cat1-GFP (pink), and cells with relatively high Cat1-GFP levels (cyan) were sorted using the single cell modus of a BD Influx sorter. (D) FACS sorting of C. albicans cells expressing relatively low or high levels of Cat1-GFP in the absence of stress (same figure as Fig 4D). (E) These FACS sorted cells (n = 200 per group) were plated onto YPD containing different concentrations of H2O2, and percentage survival (CFUs) calculated relative to the no stress control (same figure as Fig 4E). Means and standard deviations from three replicates are presented: *, p ≤ 0.05; **, p ≤ 0.01; ***, p ≤ 0.001; ****, p ≤ 0.0001. (PDF) [file ppat.1006405.s002.pdf]

Supplementary Figure 3

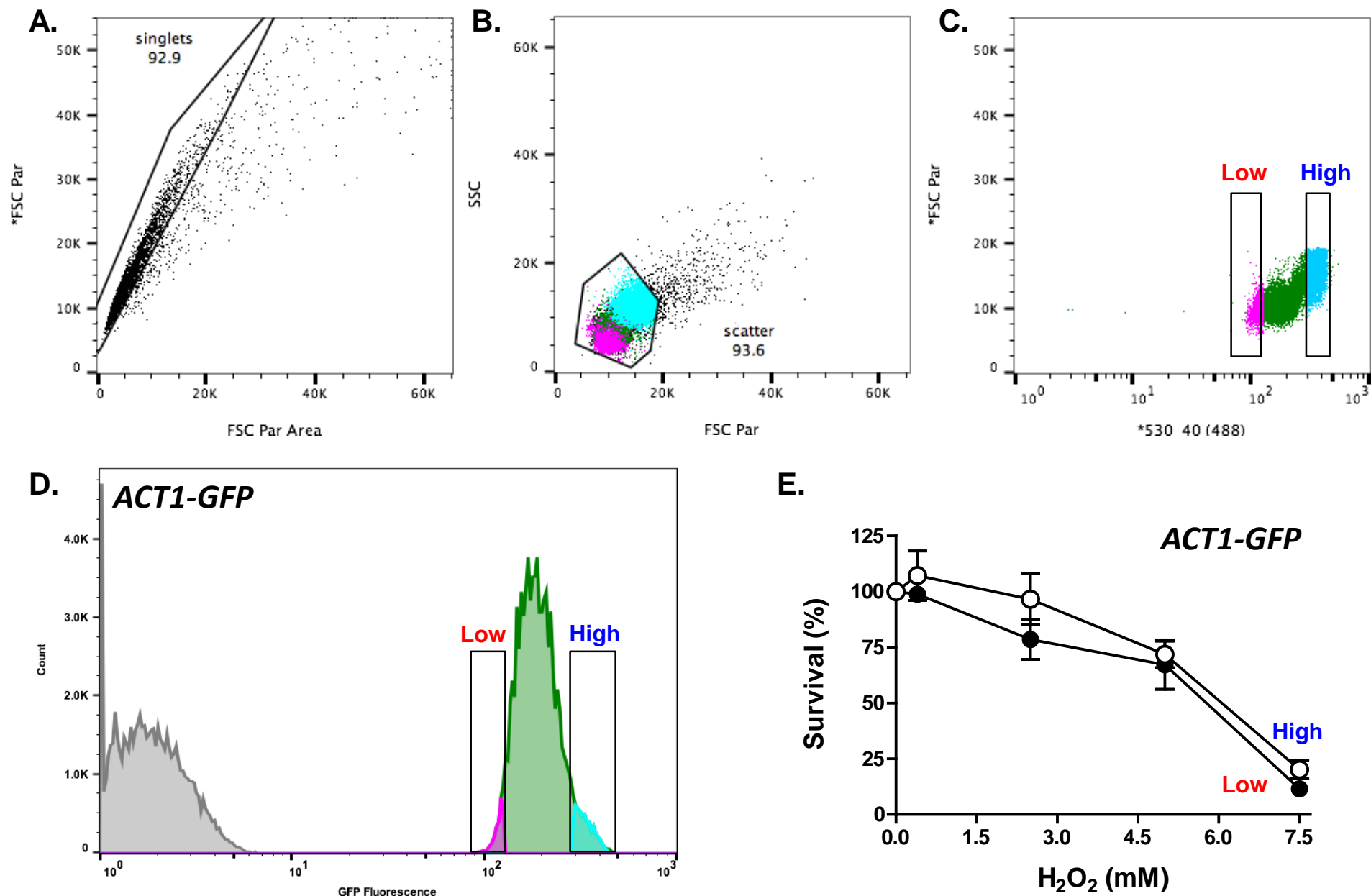

Supplement: S3 Fig — Exponential C. albicans ACT1-GFP cells (Ca230: S1 Table) were grown in the same way as for S2 Fig, and then subjected to fluorescence activated cell sorting, as before. (A) Singlets were selected and doublets excluded by analysing the FSC signals height versus area. (B) Cells of similar size were then selected by analysing the FSC versus SSC. (C) These cells were analysed for their GFP fluorescence intensity as described in S2 Fig. ACT1-GFP cells with relatively low (pink) and high GFP levels (cyan) were sorted using the single cell modus of a BD Influx sorter. (D) FACS sorting of C. albicans cells expressing relatively low or high ACT1-GFP levels in the absence of stress. (E) These FACS sorted cells (n = 200 per group) were plated onto YPD containing different concentrations of H2O2, and percentage survival (CFUs) calculated relative to the no stress control. (PDF) [file ppat.1006405.s003.pdf]

Supplementary Figure 4

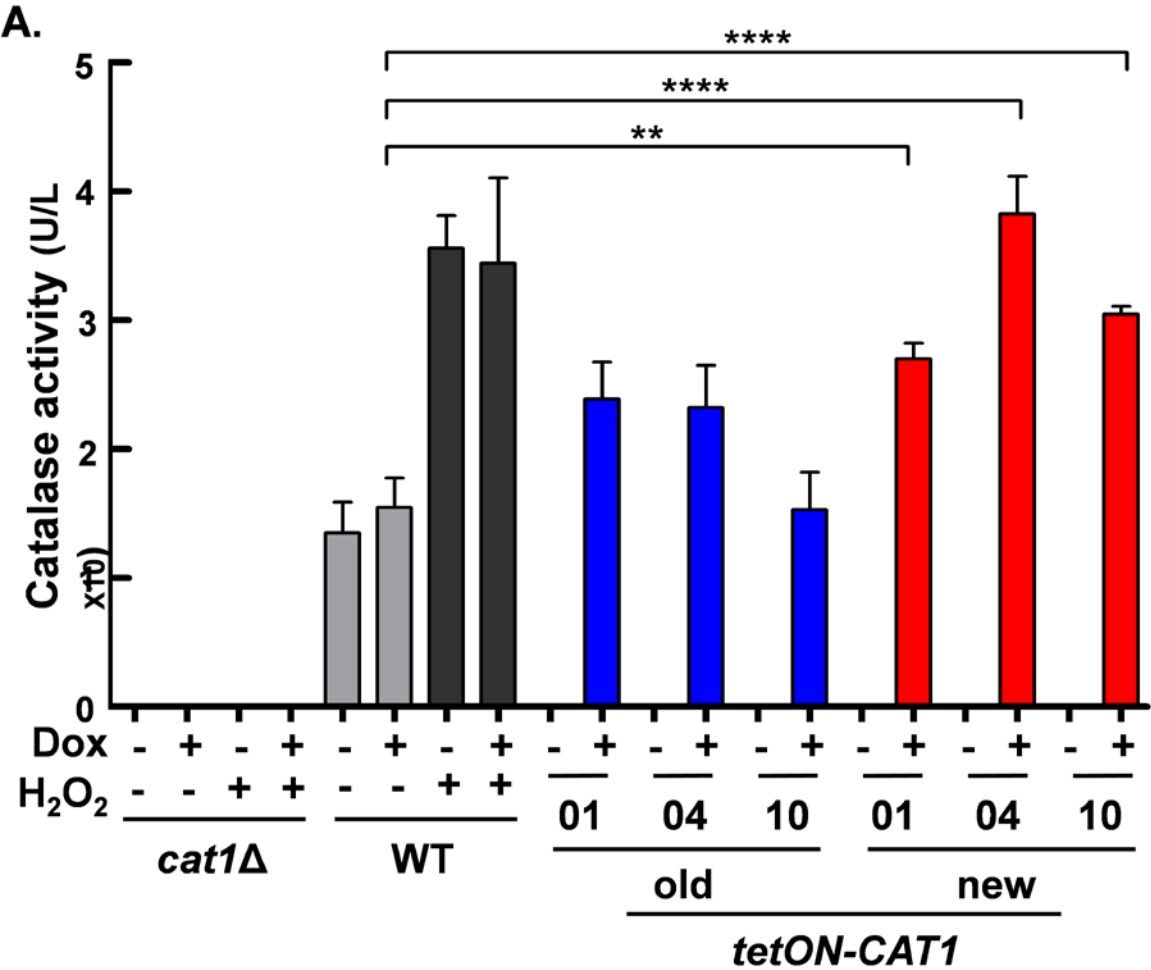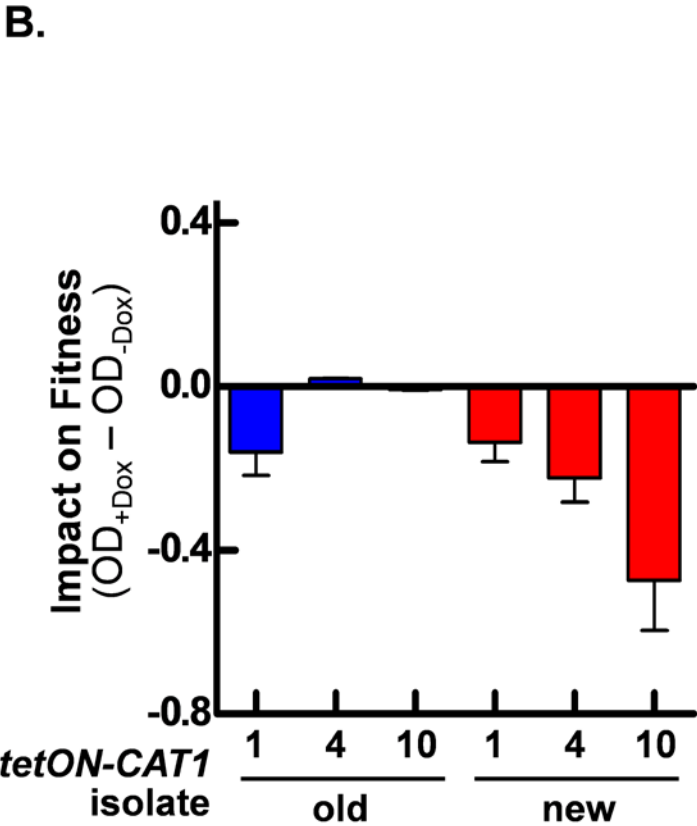

Supplement: S4 Fig — TetON-CAT1 isolates 1, 4 and 10 (Ca2038, Ca2041, Ca2044: S1 Table) behaved differently in vivo: isolate 1 displayed decreased colonisation in certain tissues (Fig 5), whereas isolates 4 and 10 did not (see text). Therefore, we tested whether isolates 4 and 10 had lost their phenotype over time. To achieve this we compared the “old” isolates 1, 4 and 10 (Ca2038, Ca2041, Ca2044) with “new” isolates (Ca2040, Ca2043, Ca2046). (Throughout this study, all experiments were performed on strains that had been freshly plated from frozen -80°C stocks. “Old” isolates were taken routinely from the same -80°C tubes over a twelve month period. These stocks were not thawed: small volumes were chipped from their frozen surface and plated. Nevertheless, the temperature of these tubes must have increased transiently from -80°C at regular intervals over this period. In contrast, “new” isolates were from identical -80°C stocks that were not touched over this period.) (A) As described in Fig 1, catalase activities were measured in C. albicans cells grown in YPD containing 0 or 20 μM doxycycline (- or + Dox, respectively): cat1Δ, Ca2089; wild-type, WT, Ca2084; blue, old tetON-CAT1 isolates, Ca2038, Ca2041, Ca2044; red, new tetON-CAT1 isolates, Ca2040, Ca2043, Ca2046 (S1 Table). Wild-type and cat1Δ cultures were exposed to 0 or 5 mM H2O2 for one hour before analysis. Means and standard deviations from three independent replicate experiments are shown, and the data were analysed using one-way ANOVA with Tukey’s post-hoc test: *, p ≤ 0.05; **, p ≤ 0.01; ***, p ≤ 0.001; ****, p ≤ 0.0001. (B) The fitness of these old and new tetON-CAT1 strains was compared in vitro by examining their growth (biomass formation; final OD600) on YPD containing or lacking doxycycline (no stress). All of the isolates displayed similar growth in the absence of doxycycline, and the wild-type (CAT1) controls remained unaffected by doxycycline. The presence of doxycycline led to decreased growth for all of the new C. a [file ppat.1006405.s004.pdf]

Supplementary Figure 5

A.

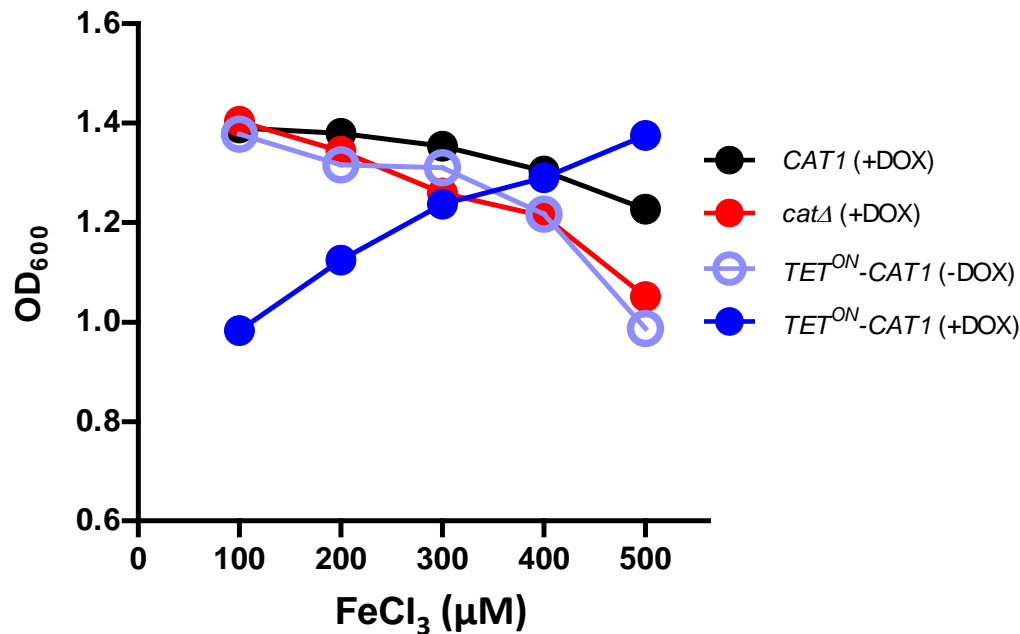

B.

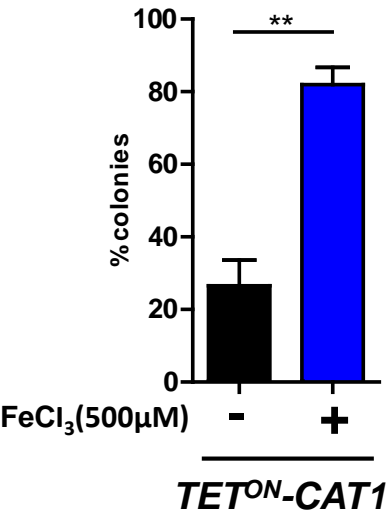

Supplement: S5 Fig — (A) Iron supplementation restores the growth of doxycycline-treated tetON-CAT1 C. albicans cells to normal, while reducing the growth of wild type and cat1Δ null cells. The growth of new C. albicans isolates was monitored (OD600) in YPD containing 0 or 20 μM doxycycline plus different concentrations of FeCl3: black circles, wild type CAT1-21 (Ca2084) cells plus doxycycline; red circles, null cat1Δ-28 (Ca2089) cells plus doxycycline; pale open circles, tetON-CAT1-1 (Ca2040) cells with no doxycycline; blue circles, tetON-CAT1-1 (Ca2040) cells with plus doxycycline (S1 Table). (B) Iron supplementation restores the ability of doxycycline-treated tetON-CAT1 cells to compete with wild type C. albicans cells in mixed cultures. Wild type (Ca2084) and tetON-CAT1-1 (Ca2040) cells were mixed in roughly equal proportions and grown for 12 h in YPD containing 20 μM doxycycline plus 0 or 500 μM FeCl3. The proportion of each cell type at the end of these competition experiments (% colonies) was determined by replica plating single colonies onto YPD plates lacking or containing combinatorial stress (1 M NaCl plus 5 mM H2O2): wild type cells are sensitive whilst tetON-CAT1 cells resistant to this stress. Means and standard deviations from three replicates are presented: *, p ≤ 0.05; **, p ≤ 0.01. (PDF) [file ppat.1006405.s005.pdf]
