# Supplementary material for: Elevated catalase expression in a fungal pathogen is a double-edged sword of iron
Source: PLoS Pathog. 2017 May 22;13(5):e1006405. doi: 10.1371/journal.ppat.1006405 (PMC5456399; doi:10.1371/journal.ppat.1006405)
Supplement: S1 Table — (PDF) [file ppat.1006405.s006.pdf]

Table S1. Strains used in this study

Strain	Name	Parent	Genotype	Source
Clinical Isolates				
SC5314		-	blood isolate	Gillum *et al.* (1984)
81/064		-	vaginal isolate	MacCallum *et al.* (2009)
AM2003/0025		-	vaginal isolate	Tavanti *et al.* (2005)
AM2005/0377		-	oral isolate	MacCallum *et al.* (2009)
SCSBB417709		-	blood isolate	Odds *et al* (2007)
J990102		-	vaginal isolate	MacCallum *et al.* (2009)
IHEM16614		-	oropharynx isolate	MacCallum *et al.* (2009)
Laboratory Strains				
CAI4		SC5314	*ura3Δ::imm434/Δura3Δ::imm434*	Fonzi *et al.* (1993)
RM1000		CAI4	*ura3Δ::imm434/Δura3Δ::imm434, his1Δ::hisG/his1Δ::hisG*	Negredo *et al.* (1997)
BWP17		RM1000	*ura3Δ::imm434/Δura3Δ::imm434, his1Δ::hisG/his1Δ::hisG, arg4Δ::hisG/arg4Δ::hisG*	Wilson *et al.* (1999)
CEC2908	*CAT1/CAT1*	BWP17	*ura3Δ::λimm434/ura3Δ::λimm434, his1Δ::hisG/his1Δ::hisG-HIS1, arg4Δ::hisG/arg4Δ::hisG-ARG4, ADH1/adh1::PTDH3-carTA::SAT1*	Chauvel *et al.* (2012)
Ca2037	*cat1Δ/cat1Δ*	CEC2908	CEC2908, *cat1Δ::loxP /cat1Δ::loxP*	This study
Ca2084	*CAT1*-21	CEC2908	CEC2908, *CAT1/CAT1, RPS1*-Clp-ptet-GTw-SP-BC21	This study
Ca2085	*CAT1*-23	CEC2908	CEC2908, *CAT1/CAT1, RPS1*-Clp-ptet-GTw-SP-BC23	This study
Ca2087	*CAT1*-26	CEC2908	CEC2908, *CAT1/CAT1, RPS1*-Clp-ptet-GTw-SP-BC26	This study
Ca2089	*cat1Δ*-28	Ca2037	CEC2908, *cat1Δ/cat1Δ, RPS1*-Clp-ptet-GTw-SP-BC28	This study
Ca2092	*cat1Δ*-38	Ca2037	CEC2908, *cat1Δ/cat1Δ, RPS1*-Clp-ptet-GTw-SP-BC38	This study
Ca2130	*cat1Δ*-54	Ca2037	CEC2908, *cat1Δ/cat1Δ, RPS1*-Clp-ptet-GTw-SP-BC54	This study
Ca2038	tetON-*CAT1-01*	Ca2037	CEC2908, *cat1Δ/cat1Δ, RPS1*-Clp-ptet-*CAT1*-GTw-SP-BC01 (old)	This study
Ca2041	tetON-*CAT1-04*	Ca2037	CEC2908, *cat1Δ/cat1Δ, RPS1*-Clp-ptet-*CAT1*-GTw-SP-BC04 (old)	This study
Ca2044	tetON-*CAT1-10*	Ca2037	CEC2908, *cat1Δ/cat1Δ, RPS1*-Clp-ptet-*CAT1*-GTw-SP-BC10 (old)	This study
Ca2040	tetON-*CAT1-01*	Ca2037	CEC2908, *cat1Δ/cat1Δ, RPS1*-Clp-ptet-*CAT1*-GTw-SP-BC01 (new)	This study
Ca2043	tetON-*CAT1-04*	Ca2037	CEC2908, *cat1Δ/cat1Δ, RPS1*-Clp-ptet-*CAT1*-GTw-SP-BC04 (new)	This study
Ca2046	tetON-*CAT1-10*	Ca2037	CEC2908, *cat1Δ/cat1Δ, RPS1*-Clp-ptet-*CAT1*-GTw-SP-BC10 (new)	This study
Ca674	RM1000+CIp20	RM1000	RIM1000, *RPS1-CIp20 (URA3,HIS1)*	Smith *et al.* (2004)
Ca1862	*CAT1/cat1Δ*	RM1000	RIM1000, *cat1::loxP-URA3-loxP/CAT1*	Kaloriti *et al.* (2014)
Ca1864	*cat1Δ/cat1Δ*	RM1000	RIM1000, *cat1::loxP-URA3-loxP/cat1::HIS1*	Kaloriti *et al.* (2014)
Ca2031	*ACT1-CAT1*	RM1000	RIM1000, *CAT1/URA3-ACT1*$_p$*-CAT1*	Kaloriti *et al.* (2014)
Ca372	CAI4+CIp10	CAI4	CAI4, *RPS1-CIp10 (URA3)*	Murad *et al.* (2000)
Ca230	*ACT1-GFP*	CAI4	CAI4, *RPS1*-pACT1-GFP	Barelle *et al.* (2004)
Ca2212		RM1000	*RIM1000, CAT1-GFP-URA3/CAT1*	This study
Ca2213	*CAT1-GFP*	RM1000	*RIM1000, CAT1-GFP-URA3/CAT1-GFP-HIS1*	This study

References

Barelle *et al.* (2004) GFP as a quantitative reporter of gene regulation in *Candida albicans. Yeast* 21, 333–340

Chauvel M *et al.* (2012) A versatile overexpression strategy in the pathogenic yeast Candida albicans: identification of regulators of morphogenesis and fitness. *PLoS ONE* 7, e45912.

Fonzi WA and Irwin MY (1993) Isogenic strain construction and gene mapping in Candida albicans. *Genetics* 134, 717-728.

Gillum AM *et al.* (1984) Isolation of the *Candida albicans* gene for orotidine-5'-phosphate decarboxylase by complementation of *S. cerevisiae ura3* and *E. coli pyrF* mutations. *Molec. Gen. Genet*. 198, 179-182.

Kaloriti D *et al.* (2014) Mechanisms underlying the exquisite sensitivity of Candida albicans to combinatorial cationic and oxidative stress that enhances the potent fungicidal activity of phagocytes. *mBio* 5, e01334-01314.

MacCallum DM, *et al.* (2009) Property differences among the four major Candida albicans strain clades. *Eukaryot Cell* 8, 373-387.

Murad, A.M.A., Lee, P.R., Broadbent, I.D., Barelle, C.J. and Brown, A.J.P. (2000) CIp10, an efficient and convenient integrating vector for *Candida albicans*. *Yeast,* 16, 325-327

Negredo A *et al.* (1997) Cloning, analysis and one-step disruption of the ARG5,6 gene of Candida albicans. *Microbiology* 143, 297-302.

Odds FC *et al.* (2007) Molecular phylogenetics of *Candida albicans*. *Eukaryot Cell* 6,1041-1052.

Smith DA *et al.* (2004) A conservedstress-activated protein kinase regulates a core stress response in the human pathogen *Candida albicans*. Mol. Biol. Cell 15, 4179–4190.

Tavanti A *et al.* (2005) Population structure and properties of *Candida albicans*, as determined by multilocus sequence typing. *J Clin Microbiol* 43, 5601-5613.

Wilson RB *et al.* (1999) Rapid hypothesis testing with Candida albicans through gene disruption with short homology regions. *J Bacteriol* 181, 1868-1874.
