# Supplementary material for: Elevated catalase expression in a fungal pathogen is a double-edged sword of iron
Source: PLoS Pathog. 2017 May 22;13(5):e1006405. doi: 10.1371/journal.ppat.1006405 (PMC5456399; doi:10.1371/journal.ppat.1006405)
Supplement: S2 Table — (PDF) [file ppat.1006405.s007.pdf]

Table S2. Primers used in this study

**Name**	**Sequence** (5'→3')	**Purpose**
ACT1 forward	ACCACCGGTATTGTTTTGGA	Quantification of the *ACT1* transcript
ACT1 reverse	AGCGTAAATTGGAACAACGTG	"
CFL5 forward	TCTGTCGCATTTGTCTCCTG	Quantification of the *CFL5* transcript
CFL5 reverse	ACAGAAATAACGTAATTCGTCAACC	"
FET3 forward	GGTGTTGATACTACCATGTTGGATT	Quantification of the *FET3* transcript
FET3 reverse	AATTGGTCCCATTGACTTGC	"
FRP1 forward	TCATGTATGGGGCGATTTCTA	Quantification of the *FRP1* transcript
FRP1 reverse	CCAACAAACAATATTCCCAACA	"
FTR1 forward	TGGTATTTCGAAACCAACACTTAC	Quantification of the *FTR1* transcript
FTR1 reverse	GGACCAGAACCGTTTTCAGA	"
cat1Δ::Clox-URA3 forward – 1st allele	TAATCATTTTAATTAAGAATATTATATTCCTTTATCAATCTCAATTGATT TAGATCTTTTTTTTATTTTCAATTTCCTATTTATATATAAATTATTTATTA CGGCCAGTGAATTGTAATA	Clox-mediated disruption of the *CAT1* locus (1st allele)
cat1Δ::Clox-URA3 reverse – 1st allele	CAATTGATGATAATCATTGCCTTACACAAAGAAGCATAAAAAAAAGCA AAACAATAGAAGAACTAATCTCAAATTAGCGCTTTCCTGGTTTCGTTC ACACTCGGAATTAACCCTCACTAA	"
cat1Δ::Clox-URA3 forward – 2nd allele	ATGGCTCCAACATTTACGAATTCTAACGGTCAACCAATTCCAGAACCA TTTGCCACTCAAAGAGTTGGTCAACACGGTCCATTGTTGTTACAAGAT TTCAACGGCCAGTGAATTGTAATA	Clox-mediated disruption of the *CAT1* locus (2nd allele)
cat1Δ::Clox-URA3 reverse – 2nd allele	TTATTTTCTTGGAGATAATTCCAAAACTTCCTTTTTGATAGCATCAGCC AAGTCTTGAGAGACTTTACCAAAGTATGCAAACACTCTGTCTTGGATA GCATCGGAATTAACCCTCACTAA	"
CAT1-GFP forward	GATGCTGCTATCCAAGACAGAGTGTTTGCATACTTTGGTAAAGTCTCT CAAGACTTGGCTGATGCTATCAAAAAGGAAGTTTTGGAATTATCTCCA AGAAAAGGTGGTGGTTCTAAAGGTGAAGAATTATT	Construction of the *CAT1-GFP* strain
CAT1-GFP-URA3 reverse	GAAAAGCAATTGATGATAATCATTGCCTTACACAAAGAAGCATAAAAA AAAGCAAAACAATAGAAGAACTAATCTCAAATTAGCGCTTTCCTGGTT TCGTTCACACTCTAGAAGGACCACCTTTGATTG	"
CAT1-GFP-HIS1 reverse	GAAAAGCAATTGATGATAATCATTGCCTTACACAAAGAAGCATAAAAA AAAGCAAAACAATAGAAGAACTAATCTCAAATTAGCGCTTTCCTGGTT TCGTTCACACGAATTCCGGAATATTTATGAGAAAC	"

Barcode forward 1	TCGTCGGCAGCGTCAGATGTGTATAAGAGACAGCGCTCTTCCGATCT CCTAGG	Primary amplification of barcodes for Barseq
Barcode reverse 1	GTCTCGTGGGCTCGGAGATGTGTATAAGAGACAGGATAGCCG CGCTGCTAGC	“
