# Supplementary material for: Elevated catalase expression in a fungal pathogen is a double-edged sword of iron
Source: PLoS Pathog. 2017 May 22;13(5):e1006405. doi: 10.1371/journal.ppat.1006405 (PMC5456399; doi:10.1371/journal.ppat.1006405)
Supplement: S3 Table — (PDF) [file ppat.1006405.s008.pdf]

Table S3. Barcodes used in this study

**Strain type**	**Barcode name**	**Barcode sequence** (5'→3')
wild-type (*CAT1*)	BC_WT_21	GCGAATGTATTGCAGCATACGGATA
wild-type (*CAT1*)	BC_WT_23	GATTAAGGATATGGGCCGACCCTTA
wild-type (*CAT1*)	BC_WT_26	ATACTTATGCACTGCTTTCGTCTTA
null mutant (*cat1*Δ)	BC_CAT1 NULL_28	TCTTTATTCTCCGCGTGGGTCATGA
null mutant (*cat1*Δ)	BC_CAT1 NULL_38	AGGCTCAAATTACGTGCCTATCGCA
null mutant (*cat1*Δ)	BC_CAT1 NULL_54	GGAGGGAAGATCGTGCGATTTAATA
tetON-CAT1	BC_TET ON_01	GTACTTATGTTACTCAGGGCGACTA
tetON-CAT1	BC_TET ON_04	CTTGCAGCGCAGTTCTCATTCGTCC
tetON-CAT1	BC_TET ON_10	GGTTCGCTGTTACTTCGGTATGTAC
